# A Refined Adaptive Laboratory Evolution Strategy With Biosensor‐Assisted Selection Resolves the Tolerance–Efficiency Trade‐Off in Toxic Chemical Biosynthesis

**DOI:** 10.1002/advs.202507740

**Published:** 2025-08-12

**Authors:** Yufei Zhang, Junhua Yun, Guoyan Zhang, Hossain M. Zabed, Yuehui Tian, Xinrui Tang, Jia Li, Xianghui Qi

**Affiliations:** ^1^ Innovative Center of Cell Signaling Transduction and Synthetic Biology,Guangdong Provincial Key Laboratory of Plant Adaptation and Molecular Design, School of Life Sciences Guangzhou University 230 Wai Huan Xi Road Guangzhou Guangdong 510006 P. R. China; ^2^ School of Food and Biological Engineering Jiangsu University 301 Xuefu Road Zhenjiang Jiangsu 212013 P. R. China

**Keywords:** 3‐hydroxypropionic acid, biosensor, adaptive laboratory evolution, mutagenesis, tolerance engineering

## Abstract

Enhancing microbial tolerance to target chemicals through conventional adaptive laboratory evolution (ALE) is time‐consuming, labor‐intensive, and further constrained by the challenge of balancing improved tolerance with maintaining optimal biosynthetic efficiency. Here, this work proposes a refined ALE strategy that combines initial mutagenesis with an automated microdroplet cultivation (MMC) system, thereby expediting the acquisition of tolerance phenotypes. Integrating a biosensor‐assisted high‐throughput screening platform enables identification of strains exhibiting advantageous “win‐win” phenotypes, characterized by simultaneous improvements in both tolerance and biosynthetic capacity. Using *E. coli* for the biosynthesis of 3‐hydroxypropionic acid (3‐HP) as a model system, this work rapidly evolves strains capable of tolerating 720 mM 3‐HP within 12 days. Leveraging a newly developed and validated 3‐HP‐responsive biosensor, this work efficiently screens and isolates superior strains. The top‐performing strain produced 86.3 g L^−1^ 3‐HP with a yield of 0.82 mol mol^−1^ glycerol. Transcriptomic analysis provide insights into mechanisms underlying this “win‐win” phenotype. Collectively, this study establishes an effective ALE framework for accelerating the development of microbial chassis tailored for high‐efficiency biochemical production.

## Graphical abstract

This study presents a refined adaptive laboratory evolution (ALE) strategy to resolve the trade‐off between tolerance and biosynthetic efficiency in toxic chemical production. Combining in vivo mutagenesis, microdroplet‐based evolution, and biosensor‐assisted selection, this strategy rapidly yields robust “win‐win” phenotypes. Applied to 3‐hydroxypropionic acid (3‐HP) production, it enables high tolerance and productivity, offering a versatile framework for microbial cell factory engineering.

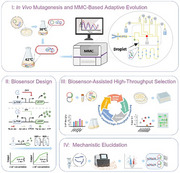

## 1 Introduction

Biomanufacturing chemicals using microbial cell factories has emerged as a sustainable and promising alternative to conventional petrochemical synthesis, aligning more closely with the principles of green chemistry and sustainable development.^[^ ^1^ ^]^ Over the past decades, metabolic engineering has played a pivotal role in enhancing the efficiency of microbial chemical production, resulting in engineered microorganisms currently utilized in industrial‐scale processes.^[^ ^2^, ^3^ ^]^ However, despite significant advancements, relying solely on metabolic engineering to rewrite and optimize the primary metabolism of chassis cells often falls short of achieving economically viable titers, rates, and yields (TRY).^[^ ^4^ ^]^ This shortfall is primarily due to physiological constraints inherent to microbial chassis that impede finely tuned biosynthetic pathways.^[^ ^5^ ^]^ One major constraint is the limited tolerance of microbial hosts to end products, such as biofuels and other biochemicals.^[^ ^6^, ^7^, ^8^, ^9^ ^]^ As these products accumulate, they exert stress on the cells, adversely affecting cellular fitness and growth, which ultimately restricts overall production TRY.^[^ ^10^ ^]^ Overcoming tolerance challenges is therefore essential to unlocking the full potential of microbial cell factories and achieving industrially relevant production levels.^[^ ^11^ ^]^

In principle, microbial chassis with enhanced tolerance to specific compounds could be rationally designed based on prior knowledge. However, in practice, this process is rarely straightforward due to the complexity of microbial metabolic and regulatory networks. Alternatively, adaptive laboratory evolution (ALE), a non‐rational engineering strategy that simulates natural evolutionary processes, is widely used to achieve robust tolerance phenotypes. ALE has proven effective in enhancing host microbial tolerance to compounds such as 1,3‐propanediol,^[^ ^10^ ^]^ ferulate,^[^ ^12^ ^]^ and isobutyl acetate,^[^ ^13^ ^]^ demonstrating its effectiveness in certain contexts. Despite its successes in many applications, traditional ALE has notable limitations. Typically, ALE begins with the pure culture of a wild‐type microbial host as the starting point for evolutionary processes.^[^ ^14^ ^]^ However, microorganisms inherently exhibit low spontaneous mutation rates, which can significantly extend the time required to observe meaningful phenotypic changes.^[^ ^15^ ^]^ These low spontaneous mutation rates can also result in evolutionary failure when desired high‐tolerance strains are not obtained. Consequently, the ALE process often becomes exceptionally time‐consuming and labor‐intensive to carry out effectively.^[^ ^16^ ^]^

Moreover, the ultimate goal of effective tolerance engineering is not only to enhance microbial resilience under high chemical concentrations but also to sustain high production metrics.^[^ ^11^ ^]^ Complicating matters further, alleviating growth inhibition does not necessarily lead to increased production. This paradox arises because, when encountering high product toxicity or environmental stress, microorganisms may redistribute internal energy and resources to prioritize survival over production.^[^ ^17^ ^]^ For instance, cells with increased tolerance may allocate more energy toward protective mechanisms such as membrane repair, antioxidant defenses, and the expression of anti‐stress proteins.^[^ ^18^ ^]^ This reallocation can diminish the energy and resources available for metabolic pathways responsible for synthesizing the target product, leading to decreased production.^[^ ^19^ ^]^ As a result, although enhanced tolerance supports survival, the added metabolic burden may hinder the overall productivity of the bioprocess.

To overcome the limitations of traditional ALE in tolerance engineering, we propose a refined strategy designed to accelerate and streamline the evolution process while overcoming the trade‐off between enhanced tolerance and sustained production capabilities. Instead of initiating ALE with a wild‐type pure culture, our approach employs a mutagenized microbial library, introducing random genomic mutations to create a diverse pool of genetic variants. This artificial enhancement of genetic diversity can increase the likelihood of beneficial mutations being present within the initial population, enabling natural selection to act immediately on advantageous variants without relying on the occurrence of spontaneous mutations. By effectively “preloading” the population with a wide spectrum of genetic possibilities, this strategy is expected to improve the probability of evolving strains capable of thriving under selective pressures, thereby enhancing the efficiency of the ALE process. Additionally, we integrate this mutagenized library with an automated microbial microdroplet culture (MMC) system and incorporate high‐throughput screening techniques.^[^ ^20^ ^]^ The MMC system enables high‐throughput cultivation of microorganisms within microliter‐scale droplets, substantially improving the scalability and efficiency of ALE experiments while minimizing resource consumption. Beyond high‐throughput cultivation, the system integrates multiple automated functions, including serial passaging, real‐time optical density monitoring, gradient‐based addition of chemical stressors, and programmable droplet sorting for the isolation of target subpopulations. This miniaturized, closed‐loop platform reduces reagent usage, manual workload, and contamination risk while enabling high‐throughput, precise, long‐term adaptive evolution. Furthermore, high‐throughput screening technology is employed to identify mutants that not only exhibit improved tolerance but also maintain or enhance production performance. This integration of mutagenized libraries, automated cultivation, and high‐throughput screening significantly reduces the time and effort typically required for ALE, while increasing the likelihood of discovering “win‐win” chassis that combine robust stress tolerance with superior production capabilities. Through this comprehensive and efficient strategy, we aim to overcome the challenges of traditional ALE and advance tolerance engineering in microbial systems.

Here, as a proof‐of‐concept, we focused on the tolerance engineering of *Escherichia coli* to enhance the biosynthesis of 3‐hydroxypropionic acid (3‐HP), serving as an example to test and validate our proposed strategy. We chose 3‐HP biosynthesis due to its designation as one of the 12 high‐value platform chemicals by the U.S. Department of Energy (DOE) and its significant industrial potential.^[^ ^21^ ^]^ However, the inherent toxicity of 3‐HP to microbial hosts has posed a critical challenge to achieving efficient production.^[^ ^22^ ^]^ Specifically, we performed in vivo mutagenesis (IVM) to generate a diverse genetic library of *E. coli* W3110,^[^ ^23^ ^]^ which served as the starting point for ALE in the MMC system. Through this approach, the evolution process was significantly accelerated and simplified. Furthermore, a biosensor‐assisted high‐throughput screening platform was established and utilized to identify individuals with superior production efficiency from the evolved populations. Notably, we obtained strains with the “win‐win” phenotype, characterized by a balance between increased tolerance and improved production capacity. Through transcriptomic sequencing, we also revealed the mechanisms contributing to enhanced 3‐HP tolerance and biosynthesis capacity, providing insights into the genetic and regulatory changes involved. In summary, our study presents a refined strategy that integrates IVM‐coupled ALE and biosensor‐assisted selection to resolve the trade‐off between microbial tolerance and production efficiency. This approach not only addresses the limitations of traditional ALE but also offers an adaptable framework for engineering microbial chassis for the efficient production of other valuable biochemicals, particularly using the versatile *E. coli* platform.

## 2 Results

### 2.1 Metabolic Engineering of the 3‐HP Biosynthetic Pathway

**Figure 1:**
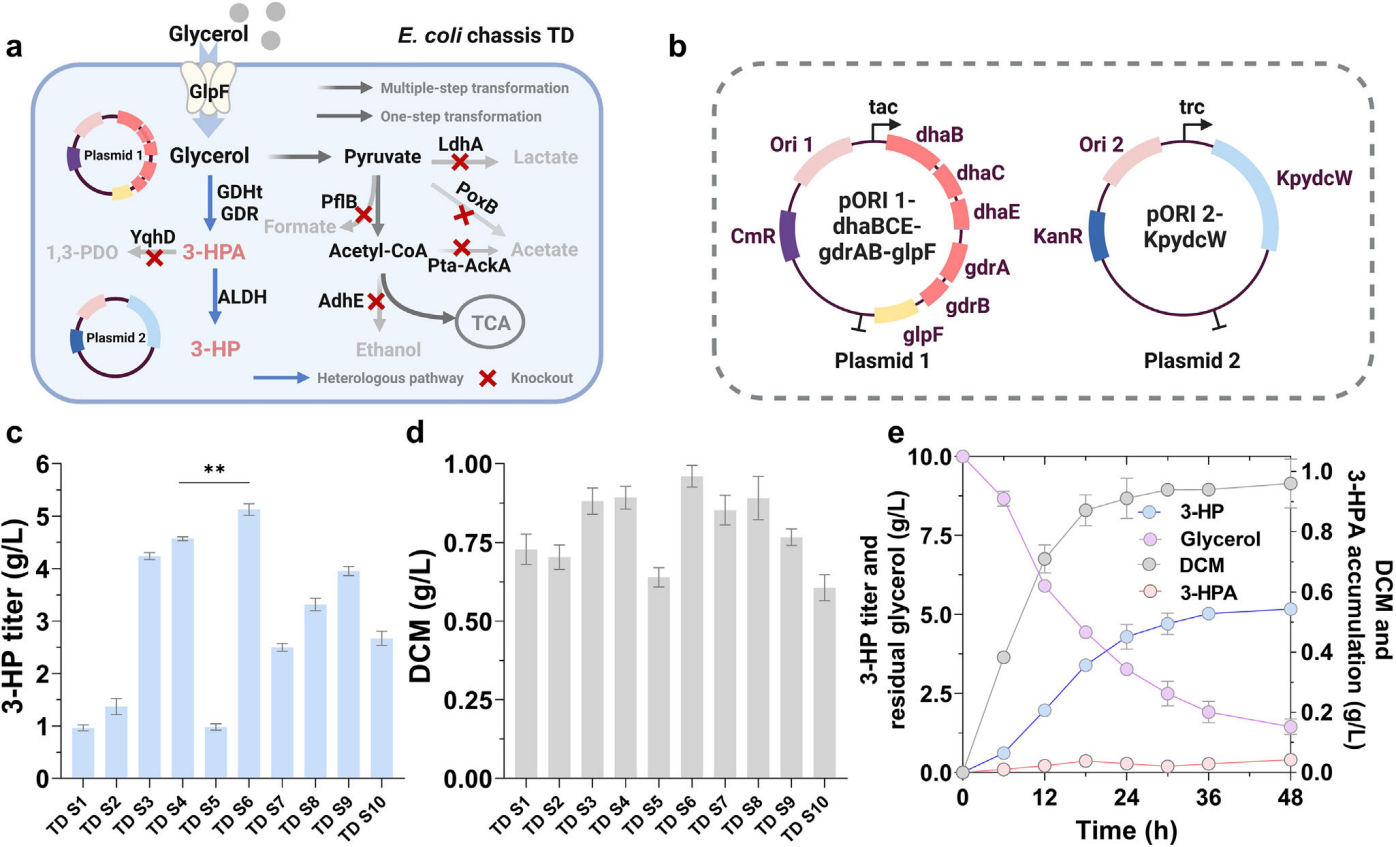
Metabolic engineering of *E. coli* W3110 for enhanced biosynthesis of 3‐HP from glycerol. a) Schematic illustration of the rewired metabolic pathway in *E. coli* chassis TD for 3‐HP biosynthesis from glycerol. b) Genetic maps of plasmid constructs. Plasmid 1 (pORI1‐*dhaBCE*‐*gdrAB*‐*glpF*) encodes the upstream module that converts glycerol to 3‐HPA, while Plasmid 2 (pORI2‐*KpydcW*) encodes an aldehyde dehydrogenase that catalyzes the downstream conversion of 3‐HPA to 3‐HP. c, d) Final 3‐HP titer and dry cell mass (DCM) of engineered strains TD S1 to TD S10 after 48 h of cultivation in modified M9 medium containing 10 g L^−1^ glycerol, 25 µM vitamin B_12_, 25 µg mL^−1^ chloramphenicol, and 50 µg mL^−1^ kanamycin (*n* = 3). e) Time‐course profile of 3‐HP titer, residual glycerol, 3‐HPA, and DCM during 48 h fermentation of strain TD S6 under the same cultivation conditions (*n* = 3). Data are presented as mean ± SD from independent biological replicates. Statistical significance was determined using a two‐tailed Student's *t*‐test, **p < 0.01. (a,b) were created in BioRender. Zhang, Y. (2025) https://BioRender.com/pfubzkv, and licensed for publication.

Pathway engineering for 3‐HP biosynthesis is a crucial foundation for subsequent tolerance engineering efforts. As an initial step, we focused on establishing a functional 3‐HP biosynthetic pathway. To this end, a tailored chassis, designated as TD, was constructed by deleting multiple genes (*adhE*, *pflB*, *ldhA*, *poxB*, *pta‐ackA*, and *yqhD*) in *E. coli* W3110 (**Figure** **1**a). These deletions targeted pathways responsible for the synthesis of major by‐products, such as ethanol, formate, lactate, acetate, and 1,3‐propanediol,^[^ ^21^ ^]^ with the goal of minimizing by‐product accumulation and streamlining metabolic flux toward 3‐HP production (Figure 1a). The 3‐HP biosynthetic pathway was recruited into chassis TD through the heterologous expression of key enzymes derived from *Klebsiella pneumoniae*, specifically glycerol dehydratase (GDHt, encoded by *dhaBCE*), its activator (GDR, encoded by *gdrAB*), and γ‐aminobutyraldehyde dehydrogenase (ALDH, encoded by *KpydcW*).^[^ ^22^ ^]^ Additionally, the glycerol facilitator (GlpF, encoded by *glpF*) was overexpressed to improve glycerol uptake (Figure 1a). The constitutive promoters tac and trc were employed to drive the expression of the *dhaBCE‐gdrAB‐glpF* cassette and *KpydcW* gene, respectively (Figure 1b). This design enabled the engineered strain to convert glycerol into the intermediate 3‐hydroxypropionaldehyde (3‐HPA) via GDHt, and then into 3‐HP through ALDH (Figure 1a). Co‐expression of GDR sustained GDHt activity, thereby enhancing the robustness and efficiency of 3‐HP biosynthesis.^[^ ^24^ ^]^

It is important to emphasize that 3‐HPA is highly cytotoxic, and its accumulation can severely impair cell growth and enzyme activity, ultimately reducing 3‐HP production.^[^ ^25^ ^]^ Therefore, rather than simply increasing the expression of both GDHt and ALDH, it is essential to achieve an appropriate balance between these two enzymes to prevent toxic buildup of 3‐HPA while ensuring efficient biosynthesis of 3‐HP. To accomplish this, we integrated the tac‐*dhaBCE‐gdrAB‐glpF* and trc‐*KpydcW* expression cassettes into plasmids with varying copy numbers. By strategically configuring different plasmid combinations (Figure S1a, Supporting Information), we fine‐tuned the 3‐HP biosynthetic pathway in chassis TD, constructing a total of 10 engineered strains (TD S1–TD S10). These strains were subjected to shake‐flask fermentation in modified M9 medium supplemented with 10 g L^−1^ glycerol. Among these strains, TD S6 exhibited superior efficiency in 3‐HP production (Figure 1c–e), achieving a titer of 5.13 g L^−1^ with negligible accumulation of 3‐HPA (Figure S1b, Supporting Information). Thus, the plasmid configuration, comprising p15A‐tac‐*dhaBCE‐gdrAB‐glpF* and pBR322‐*trc‐KpydcW*, harbored by TD S6, was selected for further applications.

### 2.2 Tolerance Engineering of Chassis TD through a Refined ALE Strategy

**Figure 2:**
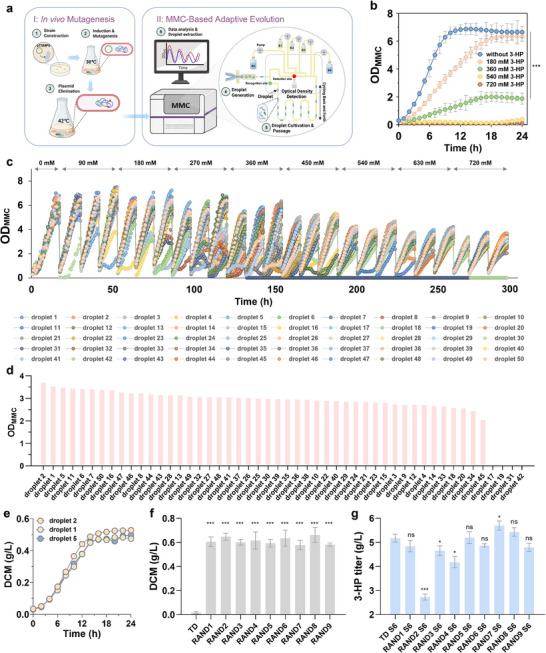
Enhancing 3‐HP tolerance of chassis TD through IVM and MMC‐based adaptive evolution. a) Schematic workflow of the IVM and MMC‐based adaptive evolution process. Step I: A mutagenized microbial library was constructed via plasmid pTSMP6‐mediated IVM. Step II: The resulting library was subjected to adaptive evolution using MMC system. In this system, bottle B0 contained the carrier oil phase; B1 and B2 supplied modified M9 medium without and with 720 mM 3‐HP, respectively; B3 held the initial mutagenized population; and B4 served as the waste reservoir. b) Sensitivity analysis of chassis TD under varying concentrations of 3‐HP in the MMC system (*n* = 5). c) Dynamic growth performance of 50 individual droplets over 300 h under incremental 3‐HP stress. d) Final optimal density (OD_MMC_) of 50 individual droplets after the ALE process. e) Shake‐flask growth curve of selected droplets (droplet 1, 2, and 5) cultivated in modified M9 medium supplemented with 720 mM 3‐HP (*n* = 3). f) Final biomass (DCM) and 3‐HP production of 9 randomly selected single colonies derived from droplet 2, with the parental chassis TD used as a control. Strains were cultivated in modified M9 medium containing 720 mM 3‐HP for 48 h (*n* = 3). g) 3‐HP production of 9 isolates derived from droplet 2 after introduction of the 3‐HP biosynthetic pathway, with strain TD S6 used as the control. Strains were cultivated in modified M9 medium containing 10 g L^−1^ glycerol, 25 µM vitamin B_12_, 25 µg mL^−1^ chloramphenicol, and 50 µg mL^−1^ kanamycin for 48 h (*n* = 3). Data are presented as mean ± SD from independent biological replicates. For two‐group comparison, statistical significance was determined using a two‐tailed Student's *t*‐test against the control group, ***p < 0.001, *p < 0.05. For multiple‐group comparison, statistical significance was determined using a one‐way ANOVA. (a) Created in BioRender. Zhang, Y. (2025) https://BioRender.com/vsoqa9m, and licensed for publication.

To enhance the 3‐HP tolerance of chassis TD, we adopted the refined ALE strategy. This approach involves utilizing a mutagenized microbial library as the initial population for ALE, promoting genetic diversity. The increased diversity allows natural selection to act immediately on advantageous variants without relying solely on spontaneous mutations, thereby facilitating the rapid acquisition of a highly tolerant phenotype. IVM technology, selected for its broad‐spectrum applicability and operational simplicity, was employed to construct the mutagenized microbial library based on chassis TD. Specifically, we employed the plasmid‐based IVM system MP6,^[^ ^23^ ^]^ which was further modified by replacing its original replicon with the thermo‐sensitive replicon pSC101(ts). This modification led to the generation of pTSMP6, a plasmid capable of efficient curing following the IVM process. Subsequently, pTSMP6 was introduced into TD prior to mutagenesis. After induction with L‐arabinose to trigger unstable replication of the genome, a population with genetic diversity was generated. By exploiting the high‐temperature‐induced instability of the replicon pSC101(ts), we cured pMP6ts and obtained the mutagenized microbial library derived from TD, designated as TD‐MUT, for subsequent ALE experiments (**Figure** **2**a).

To increase microbial cultivation throughput and reduce reliance on manual labor, we employed the automated, high‐throughput MMC system to facilitate the ALE process.^[^ ^20^ ^]^ Considering the distinct differences between the MMC system and traditional shake flask cultivation in terms of cultivation modes and optical density measurement methods, we first assessed the 3‐HP tolerance of the chassis strain TD within the MMC system to ensure its suitability. This evaluation was conducted using a “single‐factor, multi‐level” experimental framework, where 3‐HP concentration was set as the sole variable and tested across multiple levels (0, 180, 360, 540, and 720 mM). In this context, concentrations were expressed in “mM” units to facilitate convenient dilution from a 3.6 M commercial 3‐HP stock solution and to ensure precise control during ALE experiments. For reference, the tested concentrations of 180, 360, 540, and 720 mM correspond to ≈15.8, 31.6, 47.4, and 63.2 g L^−1^, respectively. Under the MMC system, the growth of TD was significantly inhibited when the concentration of 3‐HP reached 360 mM (*p* < 0.001), and nearly ceased at concentrations above 540 mM (Figure 2b). These observations were consistent with the results obtained from shake flask cultivation (Figure S2, Supporting Information), affirming the MMC system as a reliable platform for facilitating the evolution of 3‐HP tolerance.

Subsequently, TD‐MUT was employed as the initial population for the ALE process. Using the “adaptive evolution” functionality of the MMC system, TD‐MUT underwent 3‐HP tolerance evolution through automated serial passaging and continuous cultivation (Figure 2a). To maximize throughput, 50 microdroplet cultivation units (droplets 1–50) were established within the MMC system, functioning as the equivalent of 50 parallel shake‐flask cultures. These droplets were activated through a single generation of cultivation in a medium without 3‐HP and subsequently transferred to media with progressively increasing 3‐HP concentrations to initiate evolution. The 3‐HP concentration was incrementally raised from 90 to 720 mM in 90 mM steps, with three passages performed at each concentration level to allow sufficient adaptation. The entire evolution process lasted 12 days, with the growth curves of individual microdroplets shown in Figure 2c. During the activation phase of cultivation, in the absence of 3‐HP, all droplets exhibited generally favorable growth. However, notable variations in growth performance were observed among droplets. Since we used a mutagenized library rather than a pure culture as the starting point for evolution, the observed differences can be attributed to the distinct genetic characteristics of the cell populations within individual droplets, originating from TD‐MUT. During the subsequent evolution phase, as the selection pressure from 3‐HP progressively increased, the growth patterns among the droplets diverged further, particularly within the concentration range of 270 to 450 mM. Notably, this divergence was most evident in droplets such as droplet 31 and droplet 19, where cells were unable to withstand the pressure and perished entirely when the 3‐HP pressure reached 360 mM (Figure S3, Supporting Information). Such a pronounced divergence in growth trends is rarely observed when ALE is performed using pure cultures.^[^ ^26^ ^]^ Interestingly, as the 3‐HP concentration further increased to 630 mM, the differentiation in growth patterns among the surviving droplets became diminished. This observation suggests that during the ALE process, as selective pressure intensified, cell populations harboring beneficial mutations for 3‐HP tolerance managed to survive and gradually dominated within the surviving droplets. Consequently, the cells in the surviving droplets acquired superior 3‐HP tolerance and exhibited more stabilized growth patterns. After 12 days of ALE, the surviving droplets exhibited markedly enhanced tolerance to 3‐HP, demonstrating the ability to sustain growth under the selective pressure of 720 mM 3‐HP.

### 2.3 Evaluation of 3‐HP Tolerance and Biosynthesis Performance

During the final stage of adaptation to the 720 mM 3‐HP gradient, the OD_MMC_ (optical density within the MMC system) values of the surviving droplets ranged from 2.05 to 3.70 (Figure 2d). Notably, droplets 2, 1, and 5 exhibited the highest OD_MMC_ values. These droplets were subsequently extracted from the MMC system for further propagation and detailed characterization. The growth of droplets 2, 1, and 5 was initially evaluated in modified M9 medium containing 720 mM 3‐HP under shake flask conditions. The results revealed that all three droplets displayed normal growth with similar growth trends, among which droplet 2 showed a slight growth advantage (Figure 2e). To further study the properties of droplet 2, its cultivation was diluted and plated to isolate individual colonies. Nine colonies, randomly selected and designated as RAND1–RAND9, were characterized in detail to assess their 3‐HP tolerance and production capabilities. Growth assessments revealed that all tested colonies were capable of growing under 720 mM 3‐HP conditions (Figure 2f). The final biomass of the tested colonies, reflected as a dry cell mass (DCM) of ≈0.6 g L^−1^, was lower than the 1.0 g L^−1^ observed in the absence of 3‐HP (Figure S4, Supporting Information). Nevertheless, this result represented a significant improvement (*p* < 0.001) compared to the complete growth inhibition exhibited by the initial chassis TD under 720 mM 3‐HP conditions (Figure 2f). These findings suggested that cell individuals derived from droplet 2 successfully acquired the desired 3‐HP tolerance phenotype through IVM‐coupled ALE.

Subsequently, the previously constructed 3‐HP biosynthetic pathway was introduced into the isolated 3‐HP‐tolerant chassis (RAND1–RAND9), resulting in strains designated as RAND1 S6–RAND9 S6. To assess the 3‐HP biosynthesis performance of these strains, fermentations were carried out in modified M9 medium containing 10 g L^−1^ glycerol, using strain TD S6 as a control. The fermentation results, as shown in Figure 2g, revealed significant variation in 3‐HP production among the strains (*F*(8, 18) = 61.71, *p* < 0.001), with titers ranging from 2.73 to 5.70 g L^−1^. Surprisingly, most strains failed to show significant improvement in 3‐HP production compared to the control, with only RAND7 S6 achieving a notable 11.11% increase (*p* < 0.05). Among the other strains, 5 showed no significant changes (*p* > 0.05), while three exhibited markedly reduced production. In particular, RAND2 S6 exhibited the lowest 3‐HP titer at 2.73 g L^−1^, representing a 46.78% decrease compared to TD S6. The variation in 3‐HP biosynthetic performance suggested that, even after ALE, the cell population in droplet 2 remained genetically diverse, consisting of cells with varying traits. As the evolutionary direction of ALE targeted 3‐HP tolerance exclusively, most cells in this population acquired genetic mutations that improved tolerance rather than enhancing production. Additionally, certain mutations may inadvertently disrupt key metabolic pathways or regulatory networks, potentially impairing 3‐HP synthesis. These might account for the observed variations in 3‐HP production among the strains (RAND1 S6–RAND9 S6).

The aforementioned observations demonstrated the complexity of engineering microbial chassis to simultaneously achieve robust tolerance and efficient biosynthetic performance, indicating the necessity of balancing enhanced tolerance with biosynthetic efficiency. Since IVM can generate a broad spectrum of mutations, it is plausible that some cells in droplet 2 may exhibit phenotypes for both 3‐HP tolerance and high production concurrently. Given that the cells in droplet 2 already displayed a 3‐HP‐tolerant phenotype, our subsequent efforts will therefore prioritize high‐throughput screening of these individuals in droplet 2 to identify cells exhibiting a “win‐win” phenotype characterized by both high 3‐HP tolerance and elevated production.

### 2.4 Design and Modulation of a 3‐HP‐Responsive Biosensor

Due to the lack of tailored tools for high‐throughput detection of 3‐HP in microbial fermentation systems, innovative solutions are needed. Developing biosensors that can specifically respond to target metabolites and translate signals into measurable outputs, such as fluorescence or luminescence, offers a promising approach for establishing high‐throughput screening platforms. Accordingly, we aimed to design and construct a 3‐HP responsive biosensor to facilitate the identification of “win‐win” chassis with both enhanced 3‐HP tolerance and elevated production capacity.

#### 2.4.1 Identification of a 3‐HP Responsive Genetic Element

**Figure 3:**
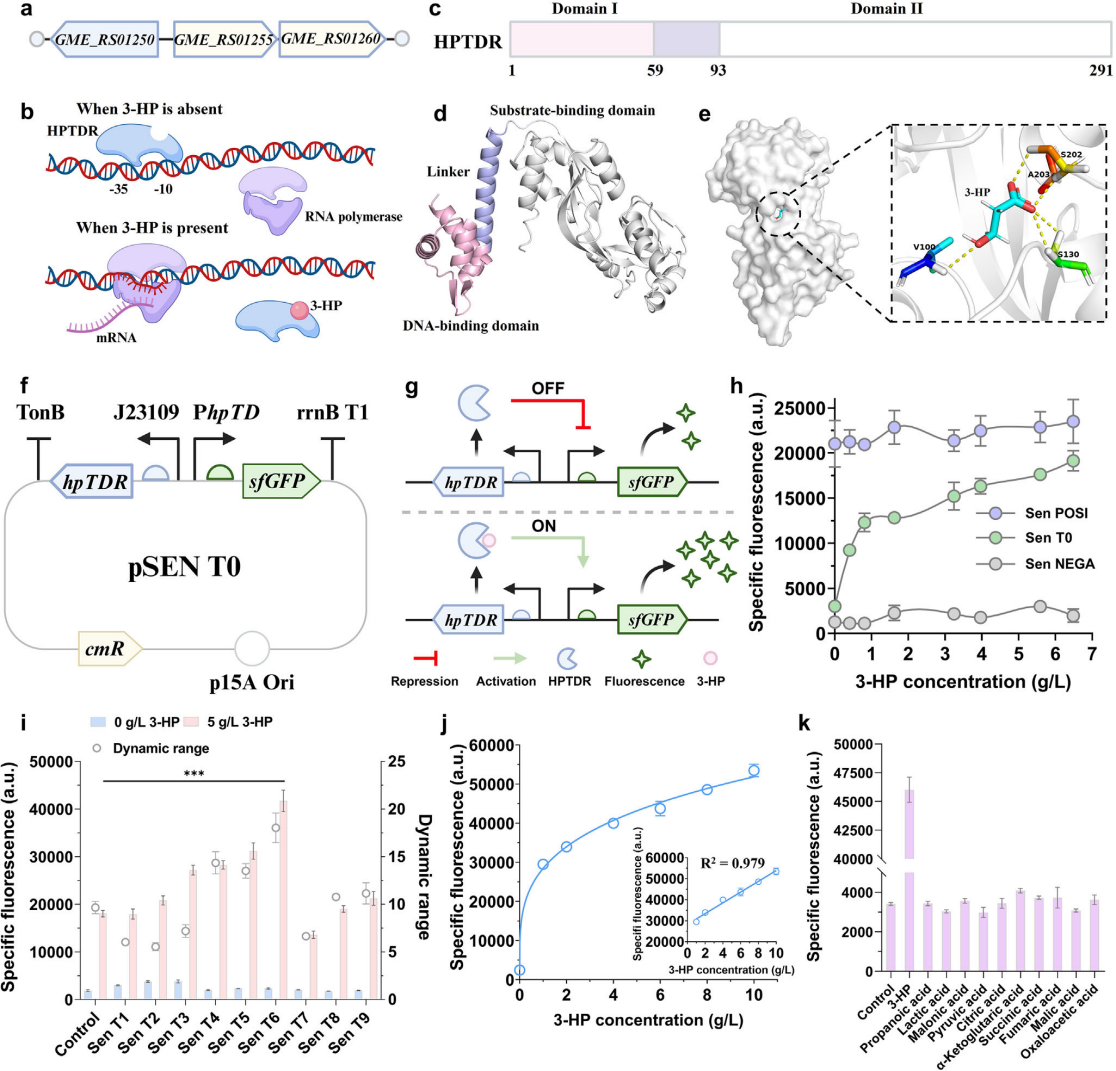
Identification, design, and optimization of a 3‐HP‐responsive biosensor. a) Genomic arrangement of *GME_RS01255*, *GME_RS01260*, and *GME_RS01250* in *H. bluephagenesis*. b) Proposed regulatory mechanism of HPTDR under the control of the P*_hpTD_* promoter, showing transcriptional repression in the absence and derepression in the presence of 3‐HP. c) Domain architecture of HPTDR, consisting of an N‐terminal DNA‐binding domain (Domain I) and a C‐terminal substrate‐binding domain (Domain II). d) Predicted 3D structure of HPTDR, with DNA‐binding and substrate‐binding regions indicated. e) Detailed view of the substrate‐binding pocket and key amino acid residues predicted to interact with 3‐HP. f) Plasmid map of the 3‐HP biosensor (pSEN T0). g) Schematic illustration of the biosensor mechanism: in the absence of 3‐HP, HPTDR binds to the promoter and represses transcription (OFF state); upon 3‐HP binding, repression is relieved, leading to sfGFP expression (ON state). h) Performance of the biosensor in response to varying concentrations of 3‐HP, measured as specific fluorescence in Sen POSI, Sen T0, and Sen NEGA. The biosensor cells were cultured in modified M9 medium supplemented with 10 g L^−1^ glycerol and 25 µg mL^−1^ chloramphenicol in microtiter plates for 9 h (*n* = 3). i) Specific fluorescence and dynamic range of biosensor variants harboring different RBS combinations (*n* = 3). j) Dose‐response curve of the optimized biosensor (pSEN T6), showing a strong positive correlation between 3‐HP concentration and specific fluorescence (*n* = 3). k) Specificity analysis of the biosensor SEN T6 in response to 3‐HP and other analogs (*n* = 3). Data are presented as mean ± SD from independent biological replicates. Statistical significance was determined using a two‐tailed Student's *t*‐test, ***p < 0.001. (b,f,g) were treated in BioRender. Zhang, Y. (2025) https://BioRender.com/o4tr10q, and licensed for publication.

In *H. bluephagenesis*, the addition of 3‐HP has been reported to activate a 3‐HP degradation pathway involving two genes, *GME_RS01255* and *GME_RS01260*.^[^ ^27^ ^]^ This activation, specifically induced by 3‐HP, suggests the existence of genetic elements in *H. bluephagenesis* that response to 3‐HP. These elements could be harnessed for the development of a 3‐HP‐responsive biosensor. Genome analysis revealed that *GME_RS01255* and *GME_RS01260* are located adjacent to each other and share a common promoter, hereafter referred to as P*_hpTD_* (Figure S5, Supporting Information). Interestingly, upstream of these two genes, another gene annotated as a LysR‐family transcription factor (LysR‐TF), GME_RS01250 (hereafter referred to as HPTDR, with its encoding gene designated as *hpTDR*), was identified (**Figure** **3**a). Notably, this gene is transcribed in the opposite direction to *GME_RS01255* and *GME_RS01260*, indicating a close genetic association and potential regulatory interaction (Figure 3a).

LysR‐TFs typically regulate genes involved in metabolism, stress responses, and biosynthesis through small‐molecule binding and conformational changes that control their activities.^[^ ^28^ ^]^ Based on this, we hypothesized that HPTDR functions as a regulatory protein. Specifically, we propose that it acts as a repressor, regulating the expression of GME_RS01255 and GME_RS01260 in response to the presence or absence of 3‐HP. In the absence of 3‐HP, HPTDR is predicted to bind to the P*_hpTD_* promoter region, blocking RNA polymerase access and silencing transcription (Figure 3b). Conversely, in the presence of 3‐HP, the molecule is speculated to interact with HPTDR, triggering a conformational change that reduces its affinity for the P*_hpTD_* promoter. This allows RNA polymerase to access the promoter, initiating the transcription and activating the 3‐HP degradation pathway (Figure 3b).

To validate this hypothesis, we conducted further bioinformatics analyses of HPTDR and investigated its interaction with 3‐HP. Protein family and domain analysis using the InterPro database confirmed that HPTDR is a typical LysR‐TF, featuring a DNA‐binding domain and a substrate‐binding domain connected by a linker peptide (Figure 3c,d). This unique structural arrangement is characteristic of LysR‐TF, which regulates gene expression by binding to small‐molecule ligands.^[^ ^29^ ^]^ Furthermore, molecular docking analysis revealed that the 3‐HP forms hydrogen bond interactions with several amino acid residues in the substrate‐binding domain of HPTDR, including Val100, Ser130, and Ala203 (Figure 3e). Together, these residuals form a stable binding pocket, ensuring the positioning of 3‐HP. This interaction likely triggers a conformational change in HPTDR, leading to its dissociation from P*_hpTD_*. These findings support our hypothesis and suggest that HPTDR and its regulatory promoter P*_hpTD_* can serve as a potential candidate for the development of a 3‐HP‐responsive biosensor.

#### 2.4.2 Design and Validation of a 3‐HP‐Responsive Biosensor

To construct a functional 3‐HP‐responsive biosensor, we employed the constitutive promoter J23129 to drive the expression of the *hpTDR* gene. This gene encodes the HPTDR protein, which serves as a key regulatory component. The green fluorescent protein gene *sfGFP*, under the control of the regulatory promoter P*_hpTD_*, served as the output signal module. These elements were assembled into the plasmid pSEN T0 (Figure 3f), with the corresponding biosensor named SEN T0. In this design, a 3‐HP input is expected to be associated with the fluorescence intensity output. Without 3‐HP, HPTDR represses P*_hpTD_*‐driven transcription, maintaining the biosensor in an “off” state with minimal fluorescence (Figure 3g). Upon 3‐HP binding, HPTDR dissociates from P*_hpTD_*, activating transcription and triggering a strong fluorescence signal, representing the “on” state (Figure 3g). To validate biosensor functionality, we also constructed two control biosensors: SEN NEGA, lacking the *sfGFP* gene, and SEN POSI, lacking the *hpTDR* gene. SEN NEGA cannot produce fluorescence, serving as the negative control. SEN POSI, devoid of HPTDR, leads to constitutive activation of P*_hpTD_* and continuous sfGFP expression, serving as the positive control.

The fluorescence outputs of SEN T0, SEN NEGA, and SEN POSI were evaluated across different 3‐HP concentrations. Specific fluorescence intensity (defined as the ratio of fluorescence to biomass) was used to normalize signal output and eliminate biomass‐related variability. As expected, SEN NEGA displayed negligible specific fluorescence intensities, attributable to background noise from the chassis or system errors (Figure 3h). SEN POSI exhibited consistently high specific fluorescence intensities (≈20 000 a.u.) across all 3‐HP concentrations, consistent with constitutive sfGFP expression due to the absence of HPTDR repression. SEN T0, in contrast, demonstrated a clear 3‐HP‐dependent response (Figure 3h). Specific fluorescence intensity increased with rising 3‐HP concentrations, indicating a strong positive correlation. Higher 3‐HP levels elicited stronger fluorescence signals, confirming that SEN T0 effectively senses 3‐HP and that HPTDR modulates P*_hpTD_*‐driven transcription in response to 3‐HP binding. This dose‐dependent fluorescence response validates the rationality of our biosensor design and underscores its potential application in quantifying environmental 3‐HP concentrations.

#### 2.4.3 Optimization and Characterization of the Designed Biosensor

The dynamic range of a biosensor is critical for accurate and efficient detection across varying target molecule concentrations.^[^ ^30^ ^]^ To optimize the dynamic range of the biosensor SEN T0, we fine‐tuned the expression levels of HPTDR and sfGFP by employing ribosome binding sites (RBS) with various strengths (B0030, B0031, and B0032). By combining these RBSs in different configurations, 9 biosensors were developed and designated as SEN T1–SEN T9 (Figure S6, Supporting Information). Specific fluorescence intensities of these biosensors were evaluated in media containing 0 and 5 g L^−1^ 3‐HP. As shown in Figure 3i, fluorescence outputs and dynamic ranges differed among the biosensors, indicating that modulation of HPTDR and sfGFP translation can profoundly influence biosensor performance. Notably, SEN T6, featuring the B0032‐*hpTDR* and B0030‐*sfGFP* combination, achieved a dynamic range of 18.03‐fold within the 0–5 g L^−1^ 3‐HP range, significantly surpassing the original biosensor SEN T0 (9.66‐fold; *p* < 0.001). We further characterized the optimized biosensor SEN T6 over an expanded 3‐HP concentration range (0–10 g L^−1^). As shown in Figure 3j, specific fluorescence intensity exhibited a strong linear relationship with 3‐HP concentration (1–10 g L^−1^). The coefficient of determination (*R^2^ =* 0.979) indicates that SEN T6 enables accurate quantitative detection within this range. The specificity of SEN T6 was assessed by measuring its response to various 3‐HP structural analogs, including propionate, lactate, malonate, pyruvate, citrate, α‐ketoglutarate, succinate, fumarate, malate, and oxaloacetate. SEN T6 exhibited high specificity for 3‐HP, with negligible responses to other tested compounds (Figure 3k). This specificity suggests the potential of SEN T6 for precise 3‐HP detection in complex fermentation environments without interference from other metabolites.

### 2.5 Biosensor‐Assisted High‐Throughput Selection

#### 2.5.1 Establishment and Validation of an In Vivo High‐Throughput Platform

**Figure 4:**
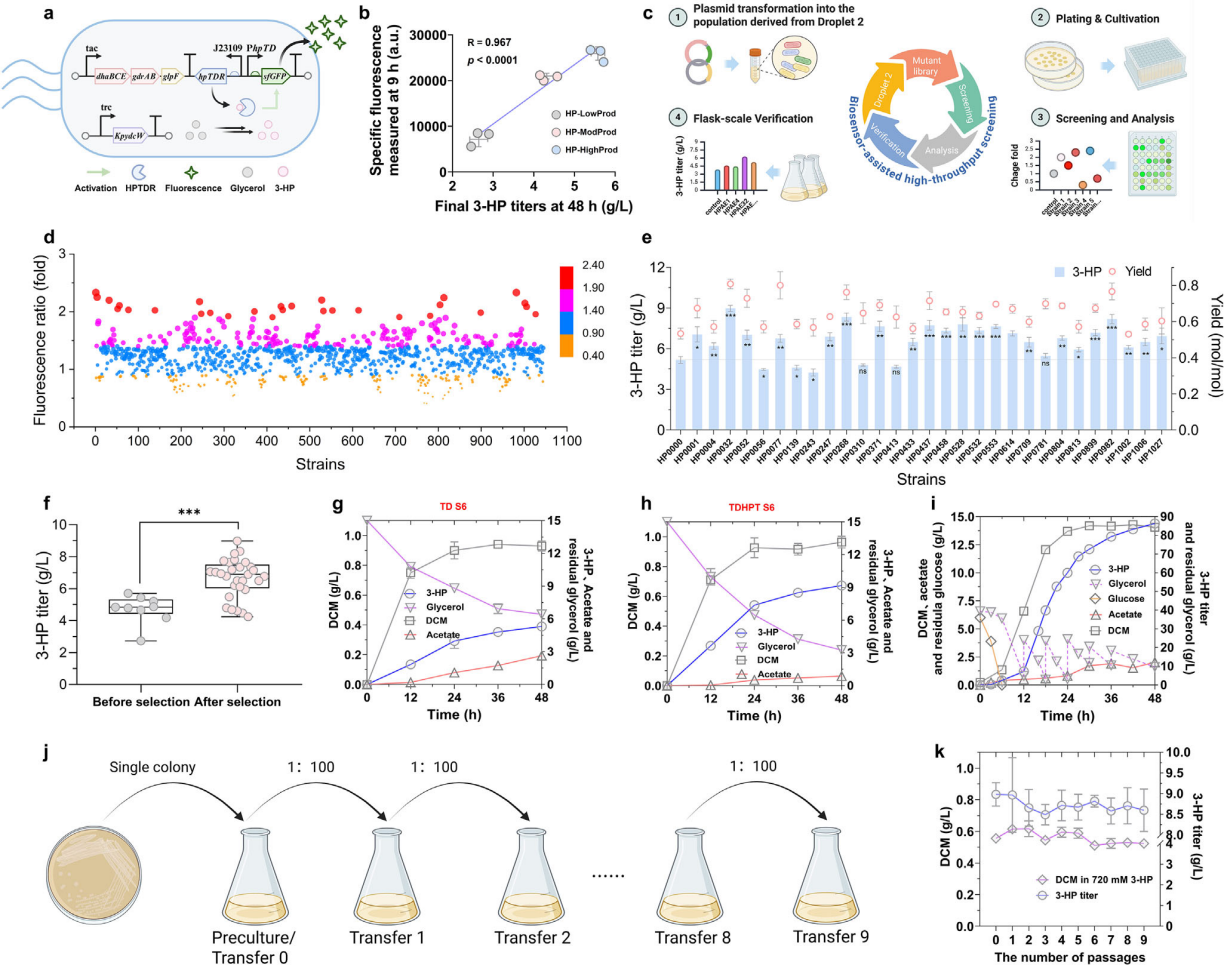
Biosensor‐assisted identification and characterization of superior 3‐HP‐producing strains derived from droplet 2. a) Schematic illustration of the biosensor‐assisted screening system, integrating genetic cassettes for 3‐HP biosynthesis and fluorescence‐based sensing. b) Correlation between specific fluorescence and final 3‐HP titers of three representative strains (HP‐LowProd, HP‐MedProd, and HP‐HighProd). Final 3‐HP titers were determined from 48 h shake‐flask fermentations, while specific fluorescence intensities were measured at 3, 9, 15, and 21 h from microplate cultures (*n* = 3). c) Diagrammatic overview of the workflow for the biosensor‐assisted screening. d) Fold change in specific fluorescence intensity relative to the control strain HP0000 among 1045 candidate strains (HP0001 – HP1045). Strains were cultivated in modified M9 medium supplemented with 10 g L^−1^ glycerol, 25 µM vitamin B_12_, 25 µg mL^−1^ chloramphenicol, and 50 µg mL^−1^ kanamycin in microplates for 9 h. e) 3‐HP production and yield of strains exhibiting > twofold changes in specific fluorescence during shake‐flask fermentation. Strains were cultured in modified M9 medium supplemented with 15 g L^−1^ glycerol, 25 µM vitamin B_12_, 25 µg mL^−1^ chloramphenicol, and 50 µg mL^−1^ kanamycin for 48 h (*n* = 3). f) Comparison of 3‐HP titers before and after selection. Prior to selection, *n* = 9 colonies were randomly picked; after selection, *n* = 29 colonies exhibiting > twofold specific fluorescence increase were evaluated (*p* < 0.0001). g, h) Time‐course profiles of 3‐HP titer, residual glycerol, DCM, and acetate in strains TD S6 and TDHPT S6. Strains were cultured in modified M9 medium containing 15 g L^−1^ glycerol, 25 µM vitamin B_12_, 25 µg mL^−1^ chloramphenicol, and 50 µg mL^−1^ kanamycin for 48 h (*n* = 3). i) Fed‐batch fermentation profile of strain TDHPT S6 (*n* = 2). j) Schematic representation of serial transfer experiments for evaluating strain stability across multiple passages. k) Stability of 3‐HP tolerance and production in strain TDHPT S6 over nine serial shake‐flask passages. Tolerance was assessed in the presence of 720 mM 3‐HP, while 3‐HP production was evaluated under standard conditions without exogenous 3‐HP (*n* = 3). Data are presented as mean ± SD from independent biological replicates. Statistical significance was determined using a two‐tailed Student's *t*‐test, *p < 0.05, **p < 0.01, ***p < 0.001. (a,c,j) were created in BioRender. Zhang, Y. (2025) https://BioRender.com/p4jpcx5, and licensed for publication.

The aforementioned experiment preliminarily validated the feasibility of the 3‐HP‐responsive biosensor through the external addition of 3‐HP in an in vitro setting. However, biosensor responses to target molecules can differ between in vitro and in vivo environments.^[^ ^31^ ^]^ To further validate the in vivo applicability of SEN T6, we integrated the biosensor within strains producing intracellular 3‐HP. For this purpose, we selected three chassis, RAND2, RAND4, and RAND7, isolated from droplet 2, based on their distinct 3‐HP production capacities. Specifically, RAND2 S6 produced 2.72 g L^−1^ of 3‐HP, RAND4 S6 achieved 4.18 g L^−1^, and RAND7 S6 reached 5.70 g L^−1^, categorizing them as low‐production (LowProd), medium‐production (MedProd), and high‐production (HighProd) chassis, respectively. To evaluate whether the biosensor could differentiate strains with varying 3‐HP production levels, the biosensor module from SEN T6 was integrated into the plasmid p15A‐tac‐*dhaBCE‐gdrAB‐glpF*, generating p15A‐tac‐*dhaBCE‐gdrAB‐glpF*‐SENSOR (**Figure** **4**a). This plasmid, along with pBR322‐trc‐*KpydcW*, was co‐transformed into RAND2, RAND4, and RAND7, resulting in strains HP‐LowProd, HP‐MedProd, and HP‐HighProd, respectively.

We first assessed the production characteristics of HP‐LowProd, HP‐MedProd, and HP‐HighProd by conducting shake‐flask fermentations and measuring the final 3‐HP titers after 48 h. The results confirmed that all three strains exhibited 3‐HP production levels comparable to those of their corresponding parental strains lacking the biosensor module (Figure 2g; Figure S7, Supporting Information). To evaluate whether the biosensor signals in microplates could reflect the final 3‐HP titers, and to determine which time point provided the most accurate correlation, we measured the specific fluorescence intensities of these strains at 3, 9, 15, and 21 h. It was shown that all four time points demonstrated a positive correlation between specific fluorescence intensities and final 3‐HP titers (Figure S8, Supporting Information). Notably, the 9‐h measurement exhibited the strongest correlation (*R* = 0.967, *p* < 0.0001), indicating that biosensor output at this stage can most accurately reflect the final production level (Figure 4b). In comparison, the 3, 15, and 21 h time points all exhibited weaker correlations (Figure S8, Supporting Information). The reduced correlation at 3 h was likely due to insufficient product accumulation, which limited biosensor activation. In contrast, the lower correlations observed at 15 and 21 h were presumably caused by signal saturation, thereby diminishing the sensor's ability to discriminate between intracellular 3‐HP levels during the later stages. Interestingly, we found that the specific fluorescence intensities measured in vivo at 9 h were lower than those obtained from in vitro with externally supplemented 3‐HP (Figure 3j; Figure S9a, Supporting Information), likely due to the fact that intracellular 3‐HP levels had not yet peaked at this early fermentation stage. Despite this, these results confirm that the biosensor can effectively distinguish strains with varying 3‐HP production capacities, demonstrating its potential as a high‐throughput platform for identifying “win‐win” individuals from the 3‐HP‐tolerant population derived from droplet 2.

#### 2.5.2 High‐Throughput Screening of Superior 3‐HP‐Producing Individuals

To identify high‐performance individuals with elevated 3‐HP production, the SEN T6 biosensor was applied in a multi‐step selection process, which included plate‐based prescreening, deep‐well plate screening, and shake‐flask verification (Figure 4c). Initially, cultures derived from droplet 2 were prepared as electrocompetent cells and transformed with plasmids p15A‐tac‐*dhaBCE‐gdrAB‐glpF*‐SENSOR and pBR322‐trc‐*KpydcW*. The transformed cells were then plated on modified M9 agar containing 10 g L^−1^ glycerol and 25 µM vitamin B_12_. After incubation, colonies with visible fluorescence were observed (Figure S9b, Supporting Information), amounting to ≈3 × 10^5^ colonies. Among these, colonies displaying strong green fluorescence were manually selected for further evaluation and transferred to LB medium to establish seed culture in 96‐well deep plates. Each plate contained 95 candidate strains and one control strain, HP0000 (chassis TD carrying the same plasmids). In total, 11 well plates, encompassing 1045 colonies (HP0001–HP1045) were processed. The selected strains were cultivated in modified M9 medium containing 10 g L^−1^ glycerol and 25 µM vitamin B_12_ in deep‐well plates. Specific fluorescence intensities were measured, and the relative fluorescence changes (HP*n*/HP0000, *n* = 1–1045) were subsequently calculated. Fluorescence intensity changes ranged from 0.42‐ to 2.43‐fold. Notably, 29 strains exhibited fluorescence intensity changes exceeding twofold (Figure 4d). These strains were selected for shake‐flask fermentation to validate their 3‐HP production capacities, with HP0000 as the control. To ensure sufficient substrate availability during fermentation, the initial glycerol concentration was increased to 15 g L^−1^. This adjustment was based on prior observations showing that 10 g L^−1^ glycerol was nearly depleted by strain TD S6 within 48 h (Figure 1e), and that the evolved strains might exhibit an enhanced capacity for glycerol utilization under the same conditions. It was shown that 23 of the 29 selected strains significantly outperformed the control (*p* < 0.05; Figure 4e). Among these, strain HP0032 exhibited the highest 3‐HP production of 8.98 g L^−1^, representing a significant increase of 74.03% compared to the 5.16 g L^−1^ observed in the control (*p* < 0.001). Moreover, HP0032 exhibited a 3‐HP yield of 0.81 mol mol^−1^ glycerol, which was the highest among all tested strains (Figure 4e).

When comparing postselection strains (HP*n* series, *n* = 1–1045) to preselection strains (HPRAND*n* series, *n* = 1–9), an increase in 3‐HP production was observed following the application of biosensor‐assisted selection (Figure 4f). Preselection strains produced 3‐HP in the range of 2.73 to 5.70 g L^−1^, with an average of 4.71 g L^−1^ and a median of 4.84 g L^−1^. In contrast, postselection strains produced 3‐HP ranging from 4.24 to 8.98 g L^−1^, with a mean of 6.65 g L^−1^ and a median of 6.89 g L^−1^. Statistical analysis confirmed that 3‐HP production was significantly enhanced after biosensor screening (*p* < 0.0001), highlighting the efficacy of biosensor‐assisted selection. However, it is also worth noting that a few selected strains exhibited lower 3‐HP titers than the control during shake‐flask validation (Figure 4e). This outcome might be attributed to the inherent genetic diversity of the evolved population derived from droplet 2, where IVM introduced a broad range of mutations. Given that the biosensor output not only depends on intracellular 3‐HP concentrations but also factors such as signal transduction efficiency and reporter expression, certain genome‐level mutations may have indirectly enhanced fluorescence without improving biosynthetic capacity. For example, mutations may have altered transcriptional regulation, increased expression resource allocation, or improved sfGFP stability, thereby elevating the signal independent of actual product formation. Consequently, some strains were prioritized during prescreening due to strong fluorescence but failed to maintain superior performance in subsequent verification. This underscores the importance of follow‐up verification steps to ensure the robustness and reliability of biosensor‐assisted selection. Furthermore, our current workflow represents a balance between screening stringency and practical considerations such as throughput, labor, and time. Specifically, although the initial plate‐based selection enabled us to visually pre‐enrich fluorescent colonies from a large population (≈3 × 10⁵), the subsequent high‐throughput screening in 96‐well deep plates was limited to 1045 isolated clones due to practical constraints. This number, despite substantial for screening, still represents a small fraction of the initial library. As a result, it is likely that some of the high‐performing strains, potentially surpassing HP0032, were not captured.

### 2.6 Evaluation of Biosynthetic Performance and Stability

To eliminate the potential metabolic burden imposed by the biosensor element, plasmids p15A‐tac‐*dhaBCE‐gdrAB‐glpF*‐SENSOR and pBR322‐trc‐*KpydcW* were cured from the selected strain HP0032 using CRISPR technology (Figure S10, Supporting Information). The resulting plasmid‐free chassis, designated TDHPT, was subsequently re‐transformed with plasmids p15A‐tac‐*dhaBCE‐gdrAB‐glpF* and pBR322‐trc‐*KpydcW*, resulting in the creation of the biosensor‐free strain TDHPT S6. Fermentation tests were then carried out in modified M9 medium supplemented with 15 g L^−1^ glycerol, with strain TD S6 serving as the control. As a result, strain TDHPT S6 achieved a 3‐HP titer of 9.17 g L^−1^ and a yield of 0.80 mol mol^−1^ glycerol, significantly surpassing the control strain (*p* < 0.001), which produced a titer of 5.31 g L^−1^ and a yield of 0.63 mol mol^−1^ glycerol (Figure 4g,h). These results represent improvements of 72.69% and 26.98% in titer and yield, respectively, for TDHPT S6 compared to the control. Meanwhile, the concentration of the byproduct acetate was significantly reduced in TDHPT S6 (*p* < 0.001), potentially contributing to the enhanced 3‐HP yield (Figure 4g,h).

To fully elucidate the potential of strain TDHPT S6, we performed fed‐batch fermentation in a 10‐L bioreactor following the protocols established in our previous study.^[^ ^22^ ^]^ The biomass of TDHPT S6 surpassed 14 g L^−1^ DCM after 48 h fermentation (Figure 4i), nearly doubling that of the control strain TD S6 (7.56 g L^−1^ DCM; Figure S11, Supporting Information). The final titer of 3‐HP in TDHPT S6 reached 86.30 g L^−1^ with a yield of 0.82 mol mol^−1^ glycerol and a productivity of 1.79 g L^−1^ h^−1^ (Figure 4i). By contrast, the control strain TD S6, under identical fermentation conditions, produced only 51.60 g L^−1^ of 3‐HP (Figure S11, Supporting Information).

Besides, we accessed the genetic stability of TDHPT S6 through successive passaging experiments (Figure 4j). After repeated passaging, strain TDHPT S6 maintained robust growth in medium containing 720 mM 3‐HP (Figure 4k). Meanwhile, the 3‐HP production capacity of TDHPT S6 under passaging conditions in the absence of 3‐HP was also evaluated. The results showed that the strain consistently produced ≈8.5 g L^−1^ of 3‐HP without significant decline in production (*p* > 0.05), indicating stable production performance over successive generations (Figure 4k). Notably, TDHPT S6 was derived from the parental strain HP0032 after elimination of endogenous plasmids and reintroduction of newly engineered plasmids. Despite extensive genetic modifications, TDHPT S6 retained the superior traits of the parental strain HP0032. These findings underscore the genetic stability and high‐performance characteristics of the evolved chassis TDHPT.

### 2.7 Transcriptome Analysis Revealing the Mechanism for Enhanced Tolerance and Biosynthesis

To investigate the genetic mechanisms underlying the enhanced tolerance and biosynthesis of 3‐HP, transcriptome sequencing was performed using RNA extracted from both the parental strain TD S6 and the evolved strain TDHPT S6 during the logarithmic phase. Comparative transcriptomic profiling identified 246 differentially expressed genes (DEGs) in TDHPT S6 relative to TD S6, applying a threshold of false discovery rate (FDR) < 0.05 and |log_2_ fold change| > 1 (Figure S12a and Table S4, Supporting Information). Among these, 28 genes were upregulated, while 218 were downregulated (Figure S12b, Supporting Information).

#### 2.7.1 The Mechanism for Enhanced 3‐HP Tolerance

Gene Ontology (GO) functional annotation of the DEGs revealed enrichment across multiple categories (Figure S13, Supporting Information). Within the “Biological process” category, terms such as “Cellular process,” “Metabolic process,” and “Response to stimulus” were notably enriched. For the “Molecular function,” prominent terms included “binding,” “catalytic activity,” and “transporter activity.” In the “Cellular component” category, “cell,” “cell part,” and “membrane” emerged as enriched terms. Notably, the enrichment of terms such as “Response to stimulus,” “Transporter activity,” “Cell part” and “Membrane” appeared to be linked to the molecular mechanisms conferring 3‐HP tolerance. Specifically, DEGs associated with “Response to stimulus” may enhance the strain's ability to adapt to environmental changes under organic acid stress conditions by regulating the expression of stress response pathways. The enrichment in “Transporter activity” suggests that membrane transport systems could play a key role in 3‐HP tolerance, potentially through the active efflux of 3‐HP or intracellular pH regulation to mitigate toxicity. Additionally, DEGs enriched in “Cell part” and “Membrane” emphasize the critical role of the cell membrane in transport and interactions with the external environment. Functional changes in the cell membrane, possibly by modulating the composition of membrane proteins and lipids, could enhance tolerance. These findings provide valuable insights into the mechanisms underlying 3‐HP tolerance.

**Figure 5:**
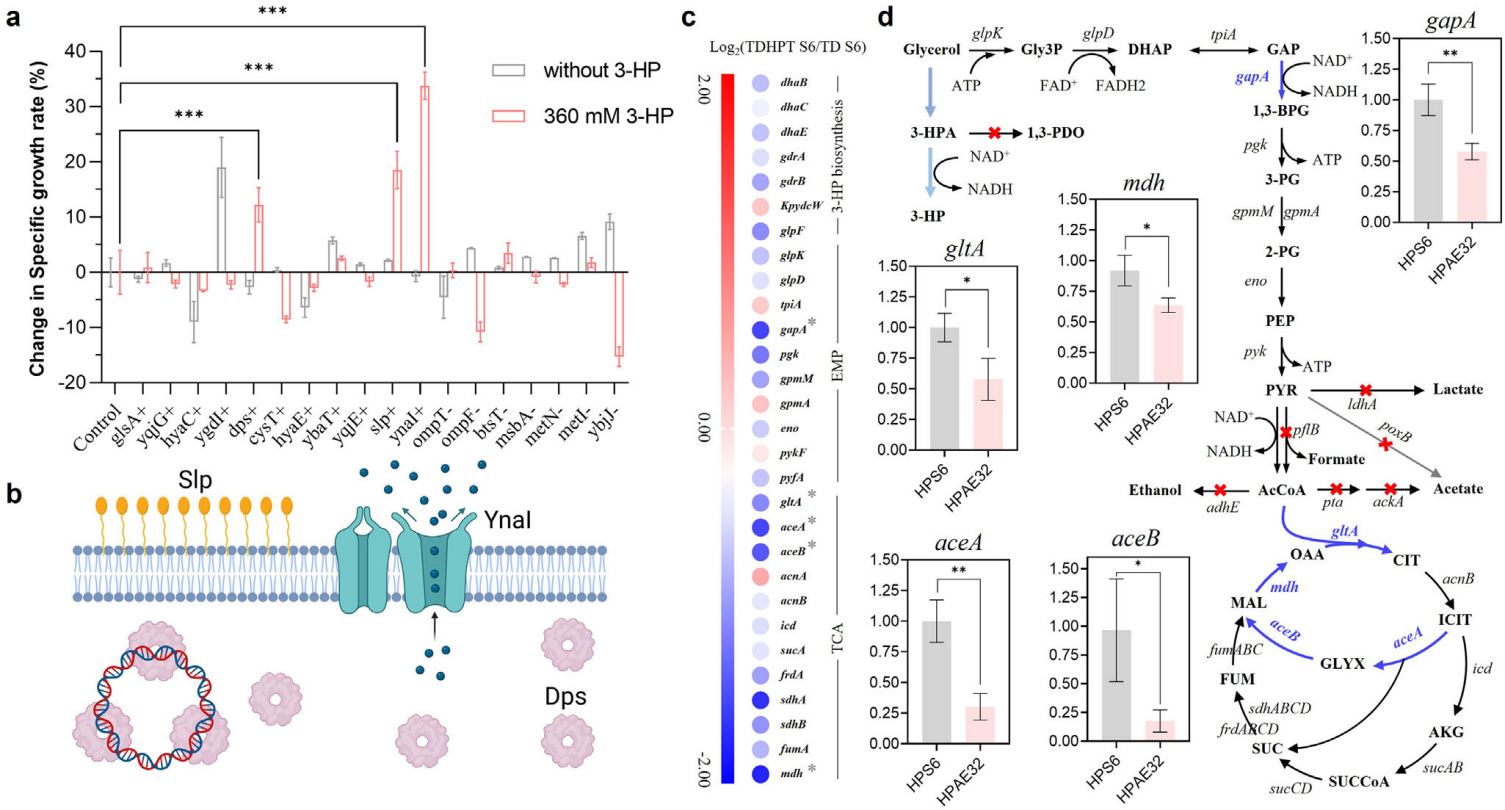
Investigation of regulatory mechanisms underlying enhanced 3‐HP tolerance and biosynthetic efficiency in evolved strain TDHPT S6. a) Effects of relevant DEG modification on strain growth rates in M9 medium with and without 360 mM 3‐HP supplementation. “+” denotes the overexpression of significantly upregulated DEGs, while “−” indicates the knockout of significantly downregulated DEGs (*n* = 3). b) Proposed mechanisms by which YnaI, Dps, and Slp contribute to improved 3‐HP tolerance. c) Transcriptomic comparison between TDHPT S6 and its parental strain TD S6, each with three biological replicates (*n* = 3), highlighting differential gene expression within the 3‐HP biosynthetic pathway, the EMP pathway, and the TCA cycle. Upregulated and downregulated genes are shown as red and blue circles, respectively; asterisks (*) denote significant DEGs (FDR < 0.05 and |log_2_ fold change| > 1). d) qPCR verification of representative DEGs involved in central metabolism, including *gapA*, *gltA*, *mdh, aceA*, and *aceB* (*n* = 3). Relative gene expression was calculated using the 2 ^–ΔΔCt^ method. A schematic overview of metabolic nodes related to glycerol utilization and central carbon metabolism is shown for reference. Abbreviations: Gly3P, glycerol‐3‐phosphate; DHAP, dihydroxyacetone phosphate, GAP, glyceraldehyde‐3‐phosphate; 1,3‐BPG, 1,3‐bisphosphoglycerate; 3‐PG, 3‐phosphoglycerate; 2‐PG, 2‐phosphoglycerate; PEP, phosphoenolpyruvate; PYR, pyruvate; AcCoA, acetyl‐CoA; CIT, citrate; ICIT, isocitrate; AKG, α‐ketoglutarate; SUC, succinate; SUC‐CoA, succinyl‐CoA; FUM, fumarate; MAL, malate; and OAA, oxaloacetate. Data are presented as mean ± SD from independent biological replicates. Statistical significance was determined using a two‐tailed Student's *t*‐test, *p < 0.05, **p < 0.01, ***p < 0.001. (b) were created in BioRender. Zhang, Y. (2025) https://BioRender.com/i43zngl, and licensed for publication.

To validate these findings, we focused on specific genes implicated in 3‐HP tolerance, as identified through transcriptomic analysis and corroborated by literature review. Eleven upregulated genes (*glsA*, *yqjG*, *hyaC*, *ygdI*, *dps*, *cysT*, *hyaE*, *ybaT*, *yqjE*, *slp*, and *ynaI*) and seven downregulated genes (*ompT*, *ompF*, *btsT*, *msbA*, *metN*, *metI*, and *ybjJ*) were selected for further functional analysis. Overexpression of the upregulated genes or knockout of the downregulated genes was performed in the parental TD strain, and specific growth rates of the resulting strains were evaluated in modified M9 medium with and without 360 mM 3‐HP. Strains carrying the empty plasmid pTrc99a served as controls. As shown in **Figure** **5**a, overexpression of the *ynaI* gene yielded a significant 33.8% increase in specific growth rate compared to the control under 360 mM 3‐HP (*p* < 0.001). The YnaI protein, a low‐conductance mechanosensitive channel, has been reported to play a vital role in cellular responses to environmental stress.^[^ ^32^ ^]^ Such channels may regulate membrane tension and facilitate the efflux of small solutes and ions,^[^ ^33^ ^]^ which could help cells adapt to volume fluctuations induced by organic acids like 3‐HP (Figure 5b). This mechanism may contribute to mitigating cellular stress and enhancing acid tolerance. In addition, overexpression of the DNA starvation/stationary phase protection protein Dps and the outer membrane lipoprotein Slp also significantly improved specific growth rates under 3‐HP exposure (*p* < 0.001). Dps is known to protect cells from oxidative damage and DNA fragmentation during environmental stress conditions.^[^ ^34^ ^]^ Under 3‐HP exposure, Dps may help safeguard genomic DNA from acid‐induced damage, thereby promoting cell survival and growth (Figure 5b). Meanwhile, Slp enhances the structural integrity of the bacterial outer membrane, serving as a protective barrier against external stressors.^[^ ^35^ ^]^ The increased membrane stability associated with Slp may help counteract the membrane‐disrupting effects of 3‐HP, thus augmenting tolerance to acidic conditions (Figure 5b). Taken together, the mechanosensitive function of YnaI, the protective role of Dps, and the structural reinforcement provided by Slp may collectively contribute to the enhanced 3‐HP tolerance observed in TDHPT S6. Nevertheless, it is important to note that other differentially expressed genes may also play contributory roles, and further systematic studies will be required to fully elucidate the genetic basis of 3‐HP tolerance.

#### 2.7.2 The Mechanism for Enhanced 3‐HP Biosynthesis Performance

In addition to the enhanced 3‐HP tolerance, strain TDHPT S6 exhibited a marked improvement in 3‐HP biosynthesis efficiency. To elucidate the mechanisms underlying this superior phenotype, we performed an analysis of the associated metabolic pathways, with a focus on the Kyoto Encyclopedia of Genes and Genomes (KEGG) enrichment results (Figure S14a, Supporting Information). The KEGG analysis identified enrichment of genes in several metabolic pathways, including “Biosynthesis of secondary metabolites,” “Arginine biosynthesis,” and “Ribosome.” Among these, “Biosynthesis of secondary metabolites” category stood out as it encompassed the largest enriched genes, indicating its potential role in driving the observed phenotypic enhancement. Interestingly, nearly all genes within this category were downregulated in TDHPT S6 compared to the parental strain (Figure S14b, Supporting Information). This finding suggested that the cells may optimize carbon allocation by downregulating secondary metabolism, thereby redirecting carbon flux away from secondary metabolite synthesis and toward the 3‐HP biosynthesis pathway. Such a reallocation of resources could enhance both the production and yield of 3‐HP synthesis, highlighting a potential adaptive mechanism for improving metabolic efficiency.

In the metabolic network of TDHPT S6, glycerol metabolism is funneled into three key pathways: 3‐HP biosynthesis, glycolysis, and the tricarboxylic acid (TCA) cycle (Figure 5c,d). Given that the transcription levels of genes directly involved in 3‐HP biosynthesis showed no significant changes (Figure 5c), our analysis focused on genes within the “Biosynthesis of secondary metabolites” category, particularly those linked to glycolysis and the TCA cycle. The results revealed that the *gapA* gene, which encodes glyceraldehyde‐3‐phosphate dehydrogenase (GapA) in the glycolytic pathway, was significantly downregulated in strain TDHPT S6 compared to the parental strain (Figure 5c). The downregulation of *gapA* may help reduce carbon loss through glycolysis, a key adjustment for optimizing carbon allocation.^[^ ^36^ ^]^ Previous studies have proved that moderate GapA activity is crucial for maintaining a balance between cell growth and the biosynthesis of target compounds.^[^ ^37^ ^]^ In our study, although *gapA* transcription was significantly reduced in TDHPT S6, the strain maintained normal growth and exhibited enhanced 3‐HP production efficiency. This observation suggests that the reduction in *gapA* transcription may not impair essential glycolytic flux required for biomass generation but instead redirect more metabolic resources toward 3‐HP biosynthesis.

Within the TCA cycle, the genes *gltA* (encoding citrate synthase) and *mdh* (encoding malate dehydrogenase), which govern the initiation and terminal steps of the cycle, respectively, were also significantly downregulated (Figure 5c,d). Concurrently, genes involved in the glyoxylate shunt, *aceA* (encoding isocitrate lyase) and *aceB* (encoding malate synthase), exhibited markedly reduced transcriptional levels (Figure 5c,d). This collective downregulation may contribute to minimizing carbon flux losses through the TCA cycle and potentially redirect carbon more efficiently toward the 3‐HP biosynthetic pathway. To independently validate and reinforce the reliability of the transcriptomic findings, we performed quantitative PCR (qPCR) analysis of these key genes associated with glycolysis and TCA cycle. This additional step was included to help address potential concerns related to platform‐specific bias or sequencing artifacts. The results showed that transcription levels of *gapA*, *gltA*, *mdh*, *aceA*, and *aceB* were downregulated by 2.61‐fold, 1.86‐fold, 1.59‐fold, 3.73‐fold, and 2.11‐fold, respectively (Figure 5d). These results were consistent with the transcriptomic data, further supporting our hypothesis. In summary, the downregulation of key genes in the “Biosynthesis of secondary metabolites” pathway, particularly those involved in glycolysis and the TCA cycle, in TDHPT S6 likely reduces carbon flux losses during glycerol metabolism. Such metabolic reprogramming is likely to contribute to minimize unnecessary energy expenditure and improve carbon utilization efficiency, thereby promoting the high‐efficiency synthesis of 3‐HP. While these findings indicate a potential metabolic shift toward enhanced 3‐HP biosynthesis, the conclusions remain correlative. In the absence of functional validation such as targeted gene perturbation or intracellular metabolite profiling, the causal relationship between transcriptional changes and the improved production phenotype cannot be definitively established. Nevertheless, the results offer valuable hypotheses that warrant further investigation.

## 3 Discussion

This study presents a refined ALE strategy that overcomes the classic trade‐off between stress tolerance and biosynthetic efficiency in microbial production of toxic chemicals. We achieved this by introducing three key modifications to conventional ALE: (i) using a pre‐mutagenized library as the starting population instead of a wild‐type strain, (ii) performing automated high‐throughput evolution in physically isolated microdroplets (MMC system), and (iii) integrating a metabolite‐responsive biosensor to couple fluorescence with biosynthetic performance. Traditional ALE typically requires several months of serial passaging and relies on the slow accumulation of rare spontaneous mutations to yield tolerant mutants.^[^ ^12^, ^15^ ^]^ In contrast, our strategy preloads genetic diversity through IVM, enabling selection to immediately act on a broader mutational landscape. By combining this diversity with automated parallel cultivation, we dramatically shortened the evolution timeline to under 2 weeks while still obtaining mutants with superior 3‐HP tolerance and production capacity. Importantly, the built‐in biosensor accelerated identification of “win‐win” variants that maintained high 3‐HP biosynthesis alongside enhanced tolerance. Overall, our integrated approach markedly improves on conventional ALE by rapidly co‐optimizing two traits (tolerance and biosynthetic efficiency) rather than each trait in isolation.

Mechanistically, pre‐evolution mutagenesis via IVM primed the cell population for accelerated adaptation by ensuring advantageous alleles were present from the outset. Once evolution commenced in the MMC system under escalating 3‐HP stress, cells carrying beneficial mutations quickly outcompeted others. Meanwhile, the 3‐HP biosensor enabled the identification of mutants with superior biosynthetic capacity by linking intracellular 3‐HP levels to fluorescence output, thereby allowing strong‐fluorescing candidates to be preferentially retained during screening. This dual selection addresses a common pitfall in tolerance evolution, where mutants often survive by diverting resources away from production.^[^ ^17^ ^]^ Instead, our “win‐win” selection pressure favored strains striking a balance between survival and biosynthesis. The outcome was a panel of evolved *E. coli* strains that not only tolerate extremely high 3‐HP concentrations (up to 720 mM) but also sustain robust 3‐HP synthesis. From a mechanistic perspective, these top‐performing mutants likely harbor combinations of mutations that mitigate 3‐HP toxicity (for instance, increasing efflux of 3‐HP) while preserving metabolic flux through the 3‐HP pathway.

Further comparative transcriptomic analysis of the parental and evolved strains provided further insight into how the trade‐off between tolerance and production was resolved. Rather than upregulating a narrow set of stress‐specific genes, the tolerant high‐producing strain exhibited broad structural and metabolic adaptations. Several genes involved in membrane structure, transport, and stress response were differentially expressed, suggesting that enhanced 3‐HP tolerance was achieved via global cell envelope remodeling and improved efflux capacity, rather than a single protective mechanism. Notably, key genes in central carbon metabolism were downregulated in the evolved strain, including *gapA*, *gltA*, and *mdh*. This shift indicates a reallocation of carbon flux: by tempering glycolysis and the TCA cycle, the cells diverted more resources toward the 3‐HP biosynthetic pathway. Similar flux redistribution has been observed in other organic acid production contexts, where reducing activity of central metabolic pathways can channel more precursors to the target product.^[^ ^36^ ^]^ Consistent with this, we also observed downregulation of genes for secondary metabolite biosynthesis in the evolved strain, suggesting a deliberate simplification of metabolism to minimize energy dissipation. The alignment between these transcriptomic changes and the improved phenotype underscores the effectiveness of our ALE strategy. It appears that evolution in our system selected for a more economized metabolic state that fortifies cellular defenses while efficiently funneling carbon to 3‐HP production. These findings not only illuminate the biological basis of the “win‐win” phenotype but can also guide rational engineering in other systems facing similar toxicity challenges. The final evolved strain (TDHPT S6) demonstrated exceptional performance in both flask and scaled fermentation trials, highlighting the practical benefits of our approach. In shake‐flask tests, TDHPT S6 grew vigorously in the presence of 720 mM 3‐HP, a concentration that completely inhibited the parental strain. This strain also substantially outperformed the unevolved strain in 3‐HP production. In 10‐L bioreactor fed‐batch fermentation, TDHPT S6 achieved a 3‐HP titer of 86.3 g L^−1^, representing an ≈67% increase over the parental strain under identical conditions, and nearly doubled the final biomass. This dramatic improvement in titer and growth demonstrates the strain's superior robustness and confirms its industrial potential for 3‐HP biomanufacturing.

Despite these encouraging outcomes, several limitations of our current strategy should be acknowledged. First, the genome‐wide mutagenesis (IVM) introduces a vast number of random mutations, some of which could inadvertently inflate the biosensor signal (e.g., by altering transcriptional regulation or improving sfGFP stability) without truly enhancing production. Such false positives might cause highly fluorescent clones to be selected even if their 3‐HP biosynthesis is not significantly improved. Although we calibrated the biosensor and confirmed product titers in shake‐flask tests, there remains a risk that certain beneficial‐looking mutations may primarily affect the sensor readout rather than the metabolic output. Second, our screening throughput of ≈10^3^ clones in deep‐well plates is modest compared to modern ultra‐high‐throughput techniques capable of processing 10⁶ to 10⁸ variants. It is likely that we sampled only a small fraction of the total mutant library, and some ultra‐rare, top‐performing variants were missed due to practical throughput limits and stringent selection thresholds. This trade‐off between screening feasibility and coverage is an inherent challenge in manual ALE workflows. Third, the context‐specific nature of evolved mutations means that certain tolerance mechanisms might not directly translate to other production pathways or host organisms. Furthermore, the use of multicopy plasmids for the biosensor and pathway genes, while convenient, can introduce variability in gene expression and metabolic burden, complicating the genotype‐phenotype relationship. These considerations underscore the importance of thorough phenotypic validation of evolved strains and point to opportunities for refining the platform.

Looking ahead, there are several avenues to enhance and generalize the refined ALE strategy. To capture a broader mutational landscape and reduce selection bias, future studies could incorporate ultra‐high‐throughput single‐cell screening technologies. For example, microfluidic droplet sorting or fluorescence‐activated cell sorting (FACS) could enable interrogation of millions of variant cells, greatly increasing the chance of isolating rare superior mutants. In parallel, full automation of the evolution and screening workflow with robotic liquid handling and multiplexed cultivation systems would facilitate large‐scale replicate ALE experiments under precisely controlled conditions. This would not only improve the statistical power of detecting true adaptive changes but also help distinguish beneficial mutations from neutral drift. The framework itself is inherently modular and can be readily extended to other target molecules. By replacing the biosensor with one based on a different transcription factor or riboswitch, the same strategy could be applied to evolve tolerance and productivity for a wide range of toxic bioproducts, including alcohols, organic acids, and aromatic compounds. Moreover, coupling our ALE approach with comprehensive systems biology analyses (e.g., whole‐genome sequencing of evolved lines, transcriptomics, proteomics, and metabolic modeling) will deepen understanding of adaptive mechanisms and reveal specific genetic targets for further rational engineering. Future implementations may also incorporate directed mutagenesis approaches, including CRISPR‐enabled genome diversification and pathway‐focused strategies, to enrich mutations within pathways known to affect tolerance or productivity and minimize the accumulation of harmful variants. Finally, systematically mapping the quantitative relationship between stress tolerance and production in diverse hosts, and scaling up this strategy to industrial bioreactors, will be important for testing its robustness and broad applicability. In summary, our refined ALE strategy provides an efficient and generalizable framework for engineering microbial cell factories that thrive under high product toxicity while maintaining high biosynthetic performance. It not only accelerates the development of robust production strains for 3‐HP, but also lays a foundation for tackling tolerance‐productivity trade‐offs in the microbial production of many other valuable yet toxic chemicals.

## 4 Experimental Section

### Reagents

The Phanta Flash Master Mix (Dye Plus) for DNA amplification and the AceQ Universal SYBR qPCR Master Mix for qPCR analysis were purchased from Vazyme Biotech (Nanjing, China). The HiScript II Q RT SuperMix for qPCR (+gDNA wiper) was also bought from Vazyme Biotech (Nanjing, China). The MultiF Seamless Assembly Mix for Gibson assembly was supplied by ABclonal Technology (Wuhan, China). QuickCut restriction endonucleases were obtained from Takara Biomedical Technology (Beijing, China). Antibiotics, including kanamycin, chloramphenicol, ampicillin, and spectinomycin, were sourced from Solarbio Science & Technology (Beijing, China). Oligonucleotide synthesis and sequencing services were provided by Azenta Life Science (Suzhou, China). 3‐HP (≈30% in water, 3.6 mol L^−1^) was acquired from TCI Development Co., Ltd. (Shanghai, China). The non‐ionic T‐F composite defoamer for fermentation was purchased from Sangon Biotech (Shanghai, China). Unless otherwise specified, additional chemicals were purchased from Sinopharm Chemical Reagent Co. Ltd. (Shanghai, China).

### Strains, Plasmids, Primers and Growth Conditions

The strains, plasmids, and primers employed in this study are presented in Tables S1–S3, Supporting Information, respectively. *E. coli* DH5α served as the cloning host, while *E. coli* W3110 was used as the starting chassis for 3‐HP biosynthesis. Plasmids pCas and pTargetF were utilized for genome editing, while the plasmid MP6 was modified to facilitate in vivo mutagenesis in *E. coli*. The plasmid pUC57Kan‐SENSOR, containing 3‐HP biosensor elements, was synthesized by Azenta. LB medium, composed of 10 g L^−1^ NaCl, 10 g L^−1^ tryptone, and 5 g L^−1^ yeast extract, was used for genetic manipulations and seed culture preparation. When necessary, the medium was supplemented with chloramphenicol (25 µg mL^−1^), kanamycin (50 µg mL^−1^), spectinomycin (50 µg mL^−1^), or ampicillin (100 µg mL^−1^).

### Plasmid and Strain Construction

The primers used for plasmid construction are listed in Table S3, Supporting Information. To construct plasmids for the constitutive expression of *dhaBCE‐gdrAB‐glpF* under the tac promoter (tac promoter sequence: 5′‐TTGACAATTAATCATCGGCTCGTATAATG‐3′), the *dhaBCE‐gdrAB‐glpF* fragment was amplified using the primers DGF F/R with the plasmid pCDF‐dhaBCE‐*gdrAB‐glpF* as the template. Simultaneously, the plasmid p15A‐tac was linearized via reverse PCR using primers backbone1 F/R. The linearized vector and amplified *dhaBCE‐gdrAB‐glpF* fragment were ligated to construct p15A‐tac‐*dhaBCE‐gdrAB‐glpF*. Subsequently, reverse PCR was performed on p15A‐tac‐*dhaBCE‐gdrAB‐glpF* using backbone2 F/R to remove the p15A origin of replication. The origins ColA, ColDF13, and pBR322 were amplified from pCOLADuet‐1, pCDFDuet‐1, and pETDuet‐1, respectively, using primers Ori1‐ColA F/R, Ori1‐ColDF13 F/R, and Ori1‐pBR322 F/R. These origins were inserted into the linearized backbone to create the plasmids pCOLA‐tac‐*dhaBCE‐gdrAB‐glpF*, pCOLDF13‐tac‐*dhaBCE‐gdrAB‐glpF*, and pBR322‐tac‐*dhaBCE‐gdrAB‐glpF*. For the expression of the *KpydcW* gene, the plasmid pRSF‐*KpydcW* served as the template, from which the *KpydcW* gene was amplified using primers *KpydcW* F/R. The plasmid pBR322‐trc was linearized with primers backbone3 F/R (trc promoter sequence: 5′‐TTGACAATTAATCATCCGGCTCGTATAATG‐3′). Ligation of the *KpydcW* gene with the linearized vector produced pBR322‐trc‐*KpydcW*. Reverse PCR with primers backbone4 F/R removed the pBR322 origin of replication. The origins p15A, ColDF13, and RSF were amplified from pACYCDuet‐1, pCDFDuet‐1, and pRSFDuet‐1, respectively, using primers Ori2‐p15A F/R, Ori2‐ColDF13 F/R, and Ori2‐pRSF F/R. These origins were inserted into the linearized backbone, yielding p15A‐trc‐*KpydcW*, pCOLDF13‐trc‐*KpydcW*, and pRSF‐trc‐*KpydcW*.

To construct the plasmid pMP6ts, the parental plasmid MP6 was digested with *Nco*I and *Xba*I, yielding a fragment containing the *araC*‐pBAD‐*dhaQ926*‐*dam‐seqA‐emrR‐ugi‐Pmcda1* cassette. To introduce a temperature‐sensitive origin, the plasmid pCas was used as a template for the amplification of the Kan^R^ and the temperature‐sensitive origin pSC101(ts) using primers Kan^R^‐TS F/R. The amplified Kan^R^‐pSC101(ts) fragment was then ligated with the *araC*‐pBAD‐*dhaQ926‐dam‐seqA‐emrR‐ugi‐Pmcda1* to create the plasmid pMP6ts.

The plasmid pSEN T0 was constructed using the synthesized plasmid pUC57Kan‐SENOR as a template to amplify the P*_hpTD_‐*P_J23109_ *‐hpTDR* fragment with primers SENSOR ELE F/R. The *sfGFP‐*rrnB T fragment was amplified from the pTrc99a*‐sfGFP* plasmid using primers *sfGFP* F/R. Additionally, the p15A origin of replication and the Cm^R^ were amplified from the plasmid pACYCDuet‐1 using primers p15A F and CmR R. These three amplified fragments were ligated to generate the plasmid pSEN T0. To construct pSEN POSI, the plasmid pSEN T0 served as a template for reverse PCR using primers backbone5 F/R, facilitating the removal of the *hpTDR* gene. The resulting PCR product was subsequently self‐ligated, producing the plasmid pSEN POSI. In a similar manner, pSEN NEGA was generated by performing reverse PCR on pSEN T0 using primers backbone6 F/R. To construct biosensor plasmids with diverse ribosome binding site (RBS) combinations, three RBS sequences were selected from the Standard Biological Parts Database (https://parts.igem.org/Ribosome_Binding_Sites/Prokaryotic/Constitutive/Community_Collection). BBa_B0031, BBa_B0032, and BBa_B0030 were chosen, exhibiting relative strengths of 0.07, 0.3, and 0.6 a.u., respectively. Using the plasmid pSEN T0 as the template, reverse PCR was performed with primers RBS1‐*hpTDR* F/R, RBS2‐*hpTDR* F/R, and RBS3‐*hpTDR* F/R to replace the original RBS of the *hpTDR* gene. The resulting fragments were self‐assembled to generate plasmids pSEN‐RBS1, pSEN‐RBS2, and pSEN‐RBS3. These plasmids were subsequently used as templates to modify the RBS of the *sfGFP* gene. Specifically, primers RBS1‐*sfGFP* F/R, RBS2‐*sfGFP* F/R, and RBS3‐*sfGFP* F/R were used to create new RBS variants for *sfGFP*. In this manner, 9 new plasmids with distinct RBS combinations were generated, designated as pSEN T1 – pSEN T9.

For the construction of plasmid p15A‐tac‐*dhaBCE‐gdrAB‐glpF*‐SENSOR, the fragment containing P_J23109_‐B0032‐*hpTDR* and P*_hpTD_*‐B0030‐*sfGFP* was amplified from plasmid pSEN T6 using primers SENSOR F/R. The plasmid p15A‐tac‐*dhaBCE‐gdrAB‐glpF* was linearized using primers backbone7 F/R. The amplified fragment was then ligated into the linearized plasmid p15A‐tac‐*dhaBCE‐gdrAB‐glpF*, yielding the final plasmid p15A‐tac‐*dhaBCE‐gdrAB‐glpF‐*SENSOR.

Using plasmid pETDuet‐1 as the template, the Amp^R^ was amplified with primers AmpR F/R. A fragment containing the temperature‐sensitive origin of replication pSC101(ts), P*_lacIq_*‐*lacI*, and sgRNA‐pBR322 was amplified from plasmid pCas using primers pSC101(ts) F and sgRNA R. Additionally, the *cas9* expression cassette was amplified from pCas with primers *cas9* F/R. The three amplified fragments were assembled to generate the plasmid pELIM‐pBR322. To construct plasmid pELIM‐p15A, using pELIM‐pBR322 as the template, reverse PCR with primers sgRNA‐p15A F/R was performed to replace the original sgRNA‐pBR322 sequence. The resulting DNA fragment was self‐ligated to yield pELIM‐p15A.

To construct plasmids for the overexpression of genes *glsA*, *yqjG*, *hyaC*, *ygdI*, *dps*, *cysT*, *hyaE*, *ybaT*, *yqjE*, *slp*, and *ynaI*, plasmid pTrc99a was linearized with primers pTrc99a F/R to generate the plasmid backbone. Genes *glsA*, *yqjG*, *hyaC*, *ygdI*, *dps*, *cysT*, *hyaE*, *ybaT*, *yqjE*, *slp*, and *ynaI* were amplified from the genomic DNA of *E. coli* W3110 using *glsA* F/R, *yqjG* F/R, *hyaC* F/R, *ygdI* F/R, *dps* F/R, *cysT* F/R, *hyaE* F/R, *ybaT* F/R, *yqjE* F/R, *slp* F/R, and *ynaI* F/R, respectively. These amplified genes were inserted into the linearized pTrc99a backbone to form pTrc99a‐*glsA*, pTrc99a*‐yqjG*, pTrc99a*‐hyaC*, pTrc99a*‐ygdI*, pTrc99a*‐dps*, pTrc99a*‐cysT*, pTrc99a*‐hyaE*, pTrc99a*‐ybaT*, pTrc99a*‐yqjE*, pTrc99a*‐slp*, and pTrc99a*‐ynaI*, respectively.

For genome‐scale gene editing, the CRISPR/Cas9 system, consisting of pCas and pTargetF, was employed. The primers and plasmids used for gene editing were detailed in Table S3, Supporting Information.

### In Vivo Mutagenesis and Adaptive Laboratory Evolution

To generate mutagenized libraries of *E. coli*, the IVM plasmid pMP6ts was utilized. The plasmid pMP6ts was first introduced into the chassis strain TD, resulting in the strain TD MP6ts. This strain was subsequently inoculated into LB medium supplemented with varying concentrations of L‐arabinose (0.01, 0.1, 1, and 10 mM). The cultures were incubated at 30 °C for 12 h. Following this initial incubation, the cultures were pooled and incubated for an additional 12 h at 42 °C to facilitate the curing of plasmid pMP6ts. After incubation, the cells were harvested by centrifugation, resuspended in 15% (v/v) glycerol, and stored at −80 °C as the TD‐MUT mutant library for future experiments.

To improve the tolerance of *E. coli* to 3‐HP, ALE was performed using the TD‐MUT library as the starting population. The ALE experiment was conducted following the guidelines of the MMC system. The preserved TD‐MUT library was diluted in modified M9 medium containing 10 g L^−1^ glycerol to an initial OD_600_ of ≈0.2, preparing it for the subsequent evolution process in the MMC system. The MMC system utilized bottles containing modified M9 medium with varying concentrations of 3‐HP: bottle No. 4 contained 0 mM, while bottle No. 6 contained 720 mM (neutralized with 10 M NaOH). Additionally, the medium supplemented with 3‐HP was sterilized through a 0.22 µm filter prior to use. The ALE process began with an 18‐h activation phase in a 3‐HP‐free medium. After this phase, eight increasing gradients of 3‐HP were established, each incremented by 90 mM. For each gradient, three successive passages were carried out at 12‐h intervals. A total of 50 microdroplet culture units were generated to facilitate synchronized evolution, with adaptive evolution conducted over a 12‐day period. At the termination of ALE, the three droplets exhibiting the highest OD_MMC_ values were individually selected and extracted. Each of these droplets was subsequently cultivated in 5 mL of modified M9 medium supplemented with 10 g L^−1^ glycerol to facilitate further analysis.

### Bioinformatics Analysis

The protein family of HPTDR was predicted using the Blast function available on the UniProt online tool (https://www.uniprot.org/blast). To further identify the domains of HPTDR, the InterPro online tool (https://www.ebi.ac.uk/interpro/) was utilized. The 3D structure of the HPTDR protein monomer was predicted using AlphaFold Server (https://alphafoldserver.com/). The 3‐HP molecule was drawn and energy‐minimized using ChemDraw and Chem3D. Subsequently, molecular docking simulations between the 3‐HP molecule and the substrate recognition domain of the HPTDR model were performed using Discovery Studio. Finally, the docking simulation results were visualized with PyMOL.

### Characterization of Biosensors

Plasmids harboring biosensor elements were introduced into the chassis *E. coli* W3110, generating strains equipped with biosensors for characterization. The resulting strains were cultivated in 96‐deep well plates, and after 9 h of incubation, samples were collected for analysis. Green fluorescence values and OD₆₀₀ values were measured using black and clear microtiter plates, respectively. Fluorescence measurements were conducted with excitation and emission wavelengths set to 485 and 530 nm, respectively.^[^ ^38^ ^]^ The specific fluorescence intensity was employed to evaluate the output strength of the biosensor's fluorescence signal, while the dynamic range served as an indicator of the biosensor's measurement capability.

(1) $$\mathrm{Specific}\;\mathrm{fluorescence}\;\mathrm{intensity}\;\left(\mathrm{SFI}\right)=\mathrm{Fluorescence}\;\mathrm{value}/OD_{600}$$

(2) $$\mathrm{Dynamic}\;\mathrm{range}=\mathrm{SF}I_{5\;g/L\;3-\mathrm{HP}}/\mathrm{SF}I_{\mathrm{without}\;3}-\mathrm{HP}$$

### Biosensor‐Assisted Selection of Superior 3‐HP Producers

The parental chassis TD and the population derived from droplet 2 were made into electrocompetent cells. The plasmids p15A‐tac‐*dhaBCE‐gdrAB‐glpF*‐SENSOR and pBR322‐trc‐*KpydcW* were co‐transformed into the prepared competent cells via electroporation. The electroporation was performed using a cuvette with a 1 mm gap under the following conditions: 1800 V, 200 Ω, and 25 µF. Cells were then incubated at 37 °C for 1 h before plating on modified M9 agar supplemented with 25 µg mL^−1^ chloramphenicol, 50 µg mL^−1^ kanamycin, 10 g L^−1^ glycerol, and 25 µM vitamin B_12_. The plates were incubated at 37 °C for 18 h. Single colonies exhibiting strong green fluorescence were selected and inoculated into a 96‐deep well plate containing LB liquid medium supplemented with 25 µg mL^−1^ chloramphenicol and 50 µg mL^−1^ kanamycin. Each plate consisted of 95 strains derived from droplet 2 and a single TD‐derived strain as a control. This procedure was repeated to generate a total of 11 deep well plates. Following a 12‐h incubation period at 37 °C, seed cultures were obtained.

Subsequently, 1% (v/v) of the seed cultures were inoculated into a new 96‐deep well plate containing modified M9 medium supplemented with 10 g L^−1^ glycerol, 25 µM vitamin B_12_, 25 µg mL^−1^ chloramphenicol, and 50 µg mL^−1^ kanamycin. Fermentation was conducted for 9 h. The green fluorescence intensity and biomass of each strain were subsequently measured. The ratio of fluorescence intensity to biomass was calculated and normalized to the control strain. Strains exhibiting fluorescence intensity ratios exceeding a twofold change relative to the control were identified for further analysis. The selected high‐performing candidates were subjected to further fermentation in modified M9 medium containing 15 g L^−1^ glycerol, 25 µM vitamin B_12_, 25 µg mL^−1^ chloramphenicol, and 50 µg mL^−1^ kanamycin. Fermentation proceeded for 48 h, after which the 3‐HP production were quantified using HPLC. The concentrations of 3‐HP and other metabolites were measured by HPLC as described earlier.^[^ ^21^ ^]^

### Elimination of Plasmids in Strain HP0032

The tool plasmid pELIMI‐p15A was transformed into strain HP0032, and the resulting strain was induced with 0.5 mM IPTG at 30 °C and 220 rpm for 12 h to cure the plasmid p15A‐tac‐*dhaBCE*‐*gdrAB*‐*glpF*‐SENSOR. The curing of pELIMI‐p15A was achieved by incubation at 42 °C for 8 h, exploiting the temperature‐sensitive instability of the replicon. Following a similar protocol, the plasmid pRB322‐trc‐*KpydcW* was subsequently cured. These successive plasmid elimination steps ultimately resulted in the generation of the plasmid‐free chassis strain, designated as TDHPT.

### Transcriptome Analysis and qPCR Verifications

The initial parental strain, TD S6, and the evolved strain, TDHPT S6, were plated on LB agar. Single colonies were selected and inoculated into modified M9 medium supplemented with 10 g L^−1^ glycerol. Cultures were incubated at 37 °C and 220 rpm until reaching the logarithmic growth phase (OD_600_ = 1.0 ± 0.05). Cells were harvested, washed twice with potassium phosphate buffer (100 mM, pH 7.0), and flash‐frozen in liquid nitrogen.

The samples were packaged in a foam‐insulated container with dry ice and dispatched to Gene Denovo Biotechnology Co., Ltd (Guangzhou, China). for RNA extraction and sequencing analysis. The transcriptome sequencing was performed on an Illumina HiseqTM 4000 sequencer by with a reference genome of *E. coli* W3110 (NC_0 07779.1). Subsequent bioinformatic analysis was performed on the web‐based toolbox “Omicsmart” (http://www.omicsmart.com). Differentially expressed genes (DEGs) were identified using a false discovery rate (FDR) < 0.05 and |log_2_ fold change| > 1 between TD S6 and TDHPT S6.

After sequencing, the RNA samples were processed for subsequent qPCR validation. Following reverse transcription with HiScript II Q RT SuperMix for qPCR (+gDNA wiper), selected cDNA targets were amplified using AceQ Universal SYBR qPCR Master Mix and the designated primers (Table S3, Supporting Information). The reactions were carried out on the StepOne Plus system (Thermo Fisher Scientific) with SYBR Green detection, employing *recA* gene as the internal reference control. Data analysis was performed according to the 2^−ΔΔCT^ method.^[^ ^39^ ^]^

### Flask and Bioreactor Fermentation for 3‐HP Production

For shake‐flask scale production of 3‐HP, single colonies of engineered strains were picked up and cultured in LB medium at 37 °C and 220 rpm for 12 h. The resulting cultivation was inoculated at 1% (v/v) ratio into a 250 mL‐flask containing 50 mL modified M9 medium. The medium composition included 1 g L^−1^ yeast extract, 0.8 g L^−1^ MgSO_4_·7H_2_O, 2 g L^−1^ NH_4_Cl, 2 g L^−1^ NaCl, potassium phosphate buffer (100 mM, pH 7.0), 5 µM vitamin B_12_, and variable concentrations of glycerol. The shake‐flask fermentations were conducted at 37 °C and 220 rpm.

For fed‐batch fermentation, the medium contained 5 g L^−1^ yeast extract, 0.8 g L^−1^ MgSO_4_·7H_2_O, 2 g L^−1^ NH_4_Cl, 2 g L^−1^ NaCl, a potassium phosphate buffer (100 mM, pH 7.0), 50 µM vitamin B_12_, 6 g L^−1^ glucose, 40 g L^−1^ glycerol, 5 mL L^−1^ trace metal solution, and 0.1% (v/v) antifoaming agent. Trace metal solution: 10 g L^−1^ FeSO_4_·7H_2_O, 2.2 g L^−1^ ZnSO_4_·7H_2_O, 2 g L^−1^ CaCl_2_, 1 g L^−1^ CuSO_4_·5H_2_O, 0.5 g L^−1^ MnSO_4_·4H_2_O, 0.1 g L^−1^ (NH_4_)_6_Mo_7_O_24_·4H_2_O, 0.02 g L^−1^ Na_2_B_4_O_7_·10H_2_O, and 0.5 M HCl.^[^ ^22^ ^]^ The fermentation was performed in a 10 L bioreactor with a working volume of 4 L. Freshly cultivated seed culture was inoculated at 10% (v/v) into the fermentation medium. Initial fermentation parameters were set as follows: temperature at 37 °C, stirring rate at 500 rpm, and an aeration rate of 1 vvm. The pH was maintained at ≈7.0 by adding aqueous ammonia (25–28%). During the fermentation process, intermittent manual feeding of a glycerol solution (containing 800 g L^−1^ glycerol and 5 g L^−1^ yeast extract) was employed to sustain the glycerol concentration at ≈20 g L^−1^. The stirring speed was automatically adjusted within a range of 500 to 1000 rpm to maintain the dissolved oxygen (DO) level above 10%. If DO levels dropped below 10% despite reaching the maximum stirring speed, the aeration rate was manually increased to 2 vvm. When excessive foaming occurred, antifoaming agent was manually added as needed. The entire fermentation process spanned 48 h.

### Statistical Analysis

Data are presented as the mean ± standard deviation (SD) from independent biological replicates (*n* = 3 unless otherwise stated). No data transformation or outlier exclusion was applied. Statistical significance was evaluated using two‐tailed Student's *t*‐tests (for two‐group comparisons) or one‐way ANOVA (for multiple‐group comparisons) in GraphPad Prism 9 (GraphPad Prism, San Diego, CA, USA), with *p <* 0.05 considered statistically significant.

## Conflict of Interest

The authors declare no conflict of interest.

## Author contributions

Y.Z., J.Y., G.Z. and H.M.Z. contributed equally to this work. Conceptualization: Y.Z., J.Y.; Methodology: G.Z., H.M.Z.; Investigation: Y.T.; Supervision: J.L., Q.X.; Writing–original draft: Y.Z.; Writing–review & editing: Q.X., Y.Z., J.Y.

## Supporting information



Supporting Information

Supplemental Table 4

## Data Availability

All data supporting the findings of this study are available in the main text or the Supporting Information files.
