# Supplementary material for: A Refined Adaptive Laboratory Evolution Strategy With Biosensor‐Assisted Selection Resolves the Tolerance–Efficiency Trade‐Off in Toxic Chemical Biosynthesis
Source: Adv Sci (Weinh). 2025 Aug 12;12(41):e07740. doi: 10.1002/advs.202507740 (PMC12591173; doi:10.1002/advs.202507740)
Supplement: Supplementary file 1 — Supporting Information [file ADVS-12-e07740-s002.docx]

**A Refined Adaptive Laboratory Evolution Strategy with Biosensor-Assisted Selection Resolves the Tolerance–Efficiency Trade-Off in Toxic Chemical Biosynthesis**

Yufei Zhang ^1,2^,† Junhua Yun ^1^,† Guoyan Zhang ^1^,† Hossain M. Zabed ^1^,† Yuehui Tian^1^, Xinrui Tang ^1,2^, Jia Li ^1^, and Xianghui Qi ^1,2^*

1 School of Life Sciences, Guangzhou University, 230 Wai Huan Xi Road, Guangzhou 510006, Guangdong, PR China

2 School of Food and Biological Engineering, Jiangsu University, 301 Xuefu Road, Zhenjiang 212013, Jiangsu, PR China.

† These authors contributed equally to this work

* Corresponding author

Email address: [qxh@gzhu.edu.cn](mailto:qxh@gzhu.edu.cn), [qxh@ujs.edu.cn](mailto:qxh@ujs.edu.cn) (X. Qi)

Tel: +86-13952892912.

**
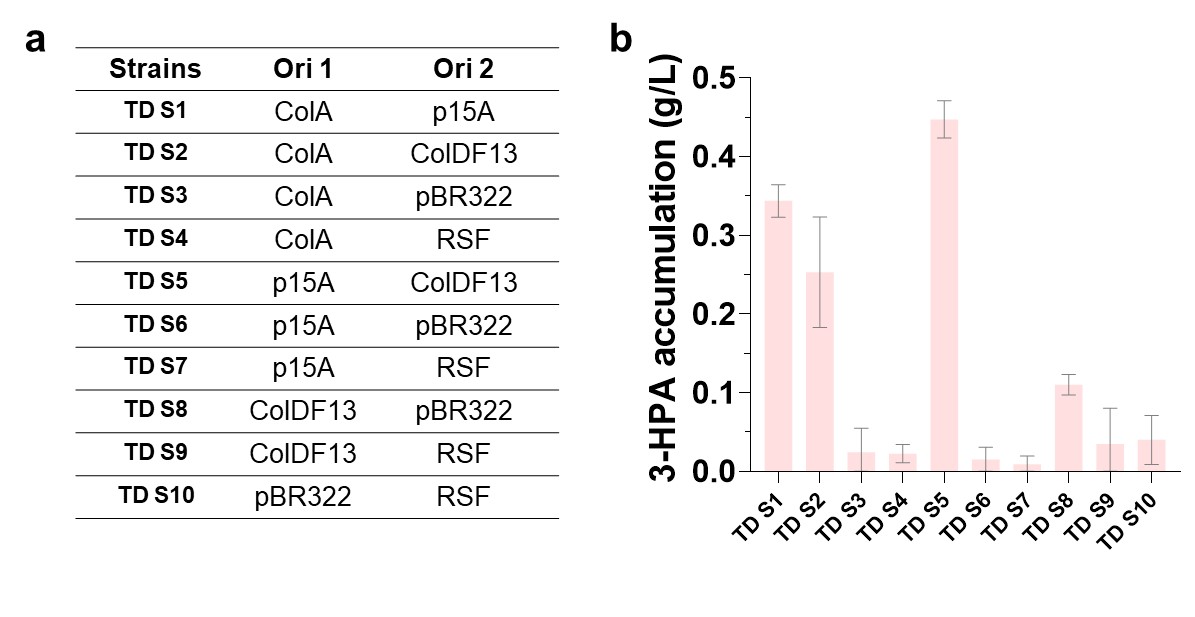
**

**Figure S1.** Optimization of 3-HP biosynthetic pathways. a) Plasmid configurations in various engineered strains. b) Accumulation of 3-HPA in the corresponding strains (*n* = 3). Data are presented as mean ± SD from independent biological replicates.

**Figure S2.** 3-HP tolerance test of the chassis TD under shake flask conditions. The experiment was conducted using a modified M9 medium supplemented with different concentrations of 3-HP (*n* = 3). Data are presented as mean ± SD from independent biological replicates.

**
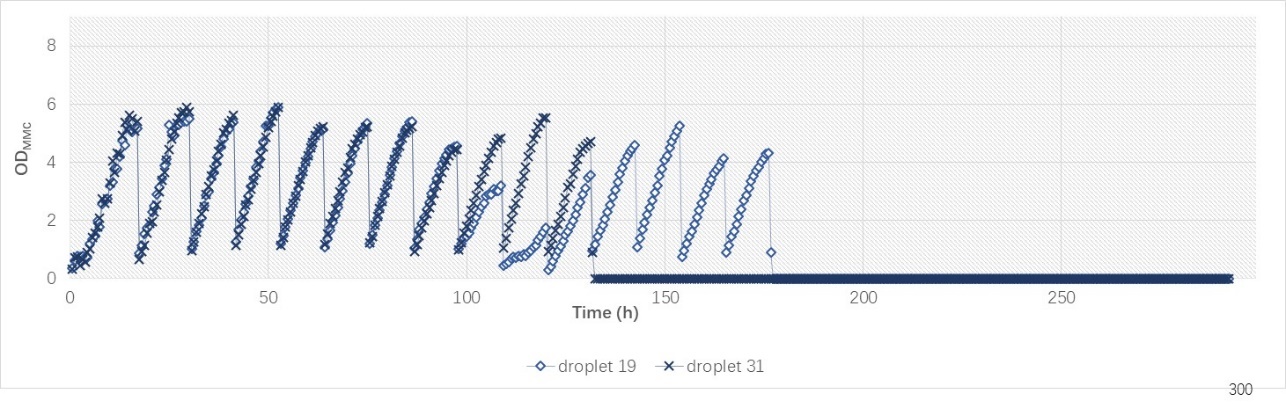
**

**Figure S3.** Growth curve of droplet 19 and droplet 31 under MMC system.

**Figure S4.** Biomass comparison of different strains (RAND1–RAND9) cultivated in modified M9 medium without 3-HP (*n* = 3). Data are presented as mean ± SD from independent biological replicates.

**
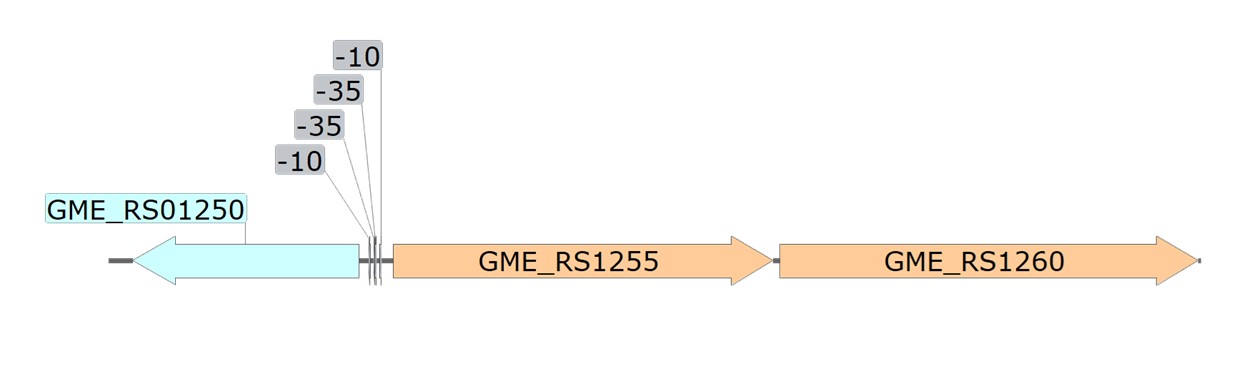
**

**Figure S5.** Schematic representation of the genetic arrangement of *GME_RS01250*, *GME_RS1255*, and *GME_RS1260*. Promoter regions with -10 and -35 consensus sequences are indicated.


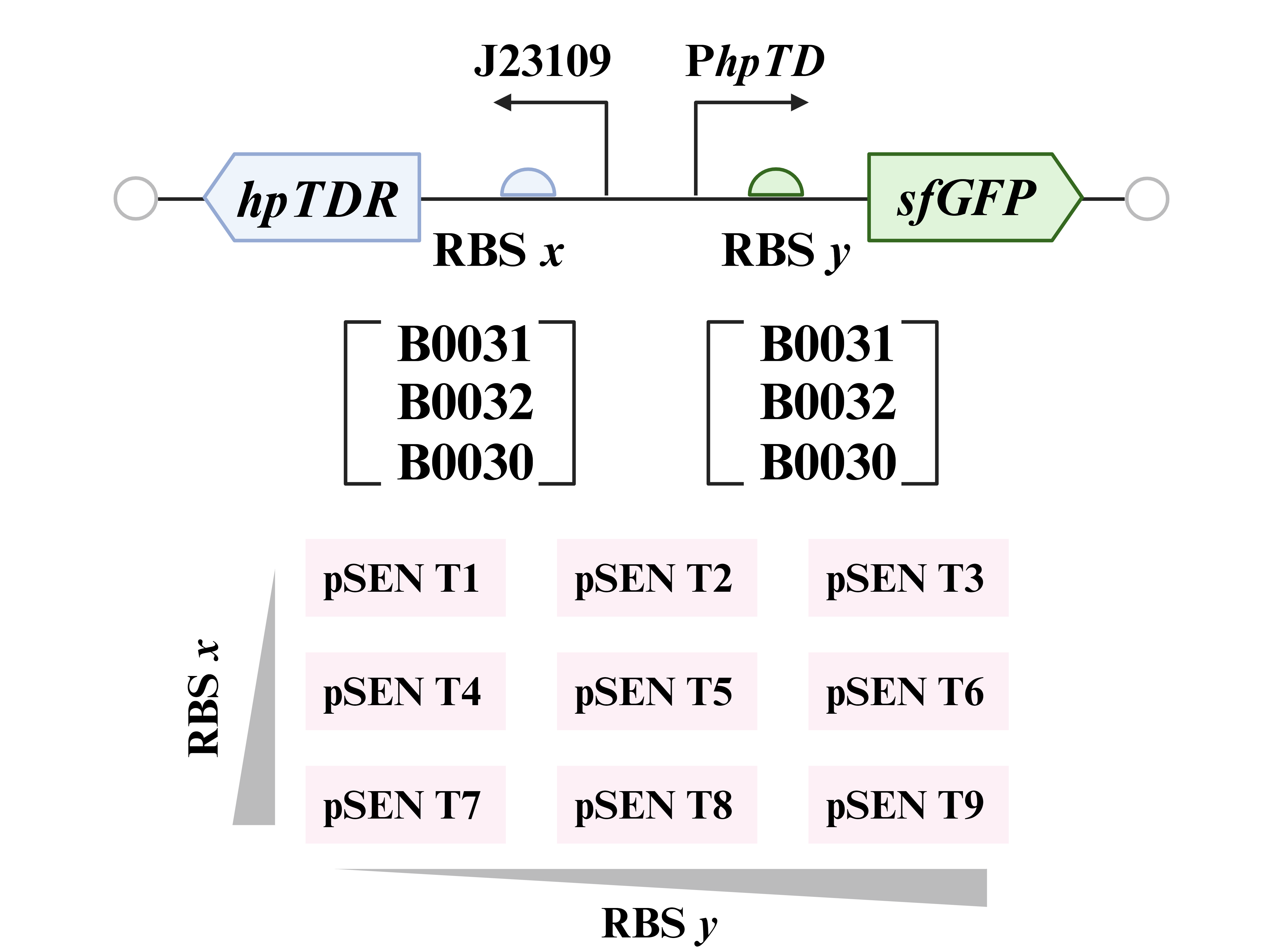


**Figure S6.** Schematic illustration of dynamic range optimization by fine-tuning the translation levels of HPTDR and sfGFP using different RBS combinations. Created in BioRender. Zhang, Y. (2025) <https://BioRender.com/i5mqigt>, and licensed for publication.

**Figure S7.** Final 3-HP titers of HP-LowProd, HP-MedProd, and HP-HighProd strains after 48 h shake-flask fermentation (*n* = 3). Data are presented as mean ± SD from independent biological replicates.


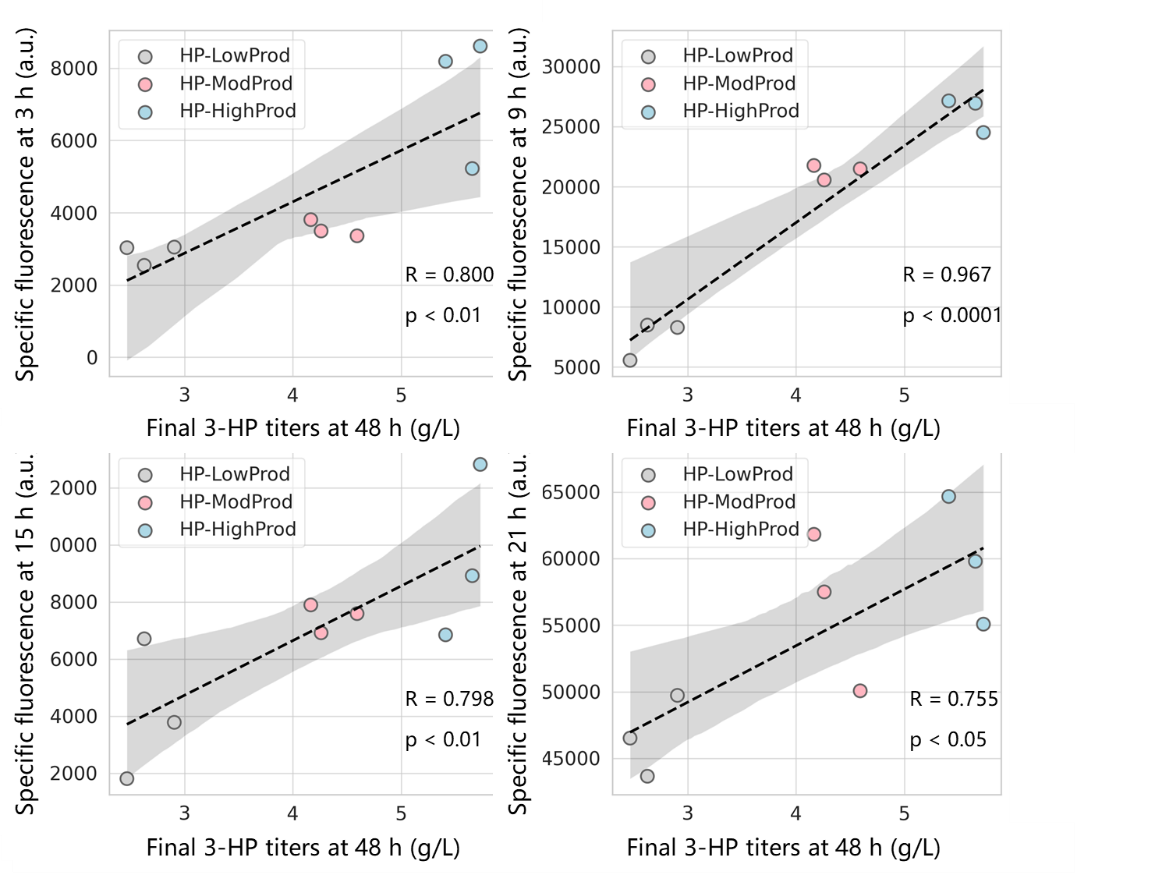


**Figure S8.** Correlation between specific fluorescence and final 3-HP titers of three representative strains (HP-LowProd, HP-MedProd, and HP-HighProd). Final 3-HP titers were determined from 48 h shake-flask fermentations, while specific fluorescence intensities were measured at 3, 9, 15, and 21 h from microplate cultures (*n* = 3). Shaded areas represent 95% confidence intervals for the regression fit.


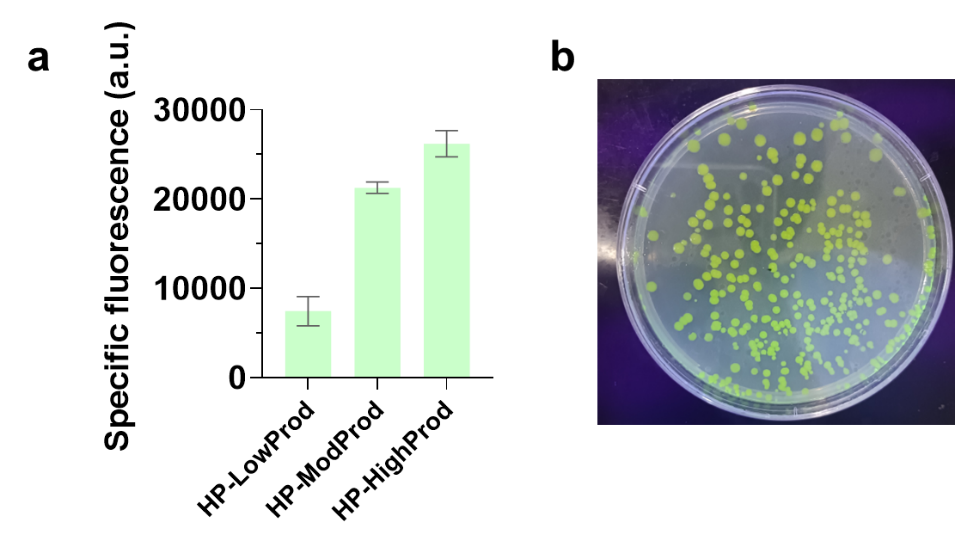


**Figure S9.** Visualization of green fluorescence in engineered strains. a) Fluorescence of cell pellets after 9 h cultivation (*n* = 3). b) Fluorescent colonies grown on modified M9 agar plates after 18 h incubation. Transformed cells were plated on modified M9 medium supplemented with 10 g/L glycerol, 25 µM vitamin B₁₂, 25 μg/mL chloramphenicol, and 50 μg/mL kanamycin. Data are presented as mean ± SD from independent biological replicates.


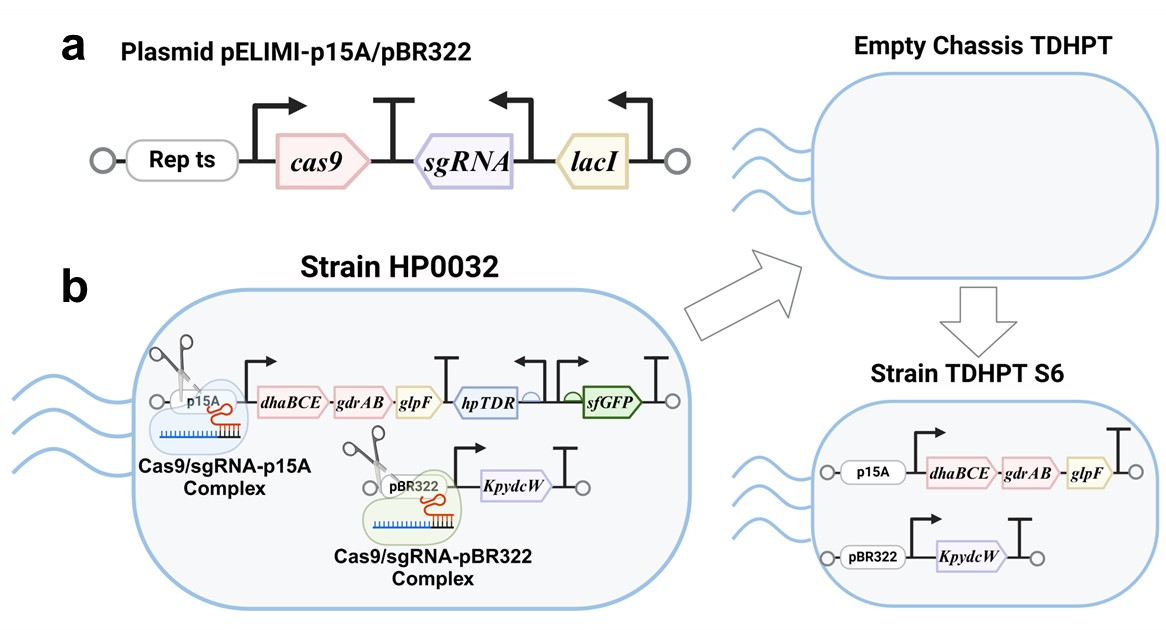


**Figure S10**. Elimination of plasmids in the evolved strain HP0032. a) Schematic representation of the plasmid map for the tool plasmid pELIMI-p15A/pBR322. b) Procedure for plasmid elimination and strain construction. Created in BioRender. Zhang, Y. (2025) <https://BioRender.com/fu2y5jv>, and licensed for publication.

**Figure S11.** Fed-batch fermentation profile for strain TD S6 (*n* = 2). The fermentation was performed in a 10 L bioreactor with a working volume of 4 L. Freshly cultivated seed culture was inoculated at 10% (v/v) into the fermentation medium. Initial fermentation parameters were set as follows: temperature at 37°C, stirring rate at 500 rpm, and an aeration rate of 1 vvm. The pH was maintained at approximately 7.0 by adding aqueous ammonia (25–28%). During the fermentation process, intermittent manual feeding of a glycerol solution (containing 800 g/L glycerol and 5 g/L yeast extract) was employed to sustain the glycerol concentration at approximately 20 g/L. The stirring speed was automatically adjusted within a range of 500 to 1000 rpm to maintain the dissolved oxygen (DO) level above 10%. If DO levels dropped below 10% despite reaching the maximum stirring speed, the aeration rate was manually increased to 2 vvm. When excessive foaming occurred, antifoaming agent was manually added as needed. The entire fermentation process spanned 48 h.


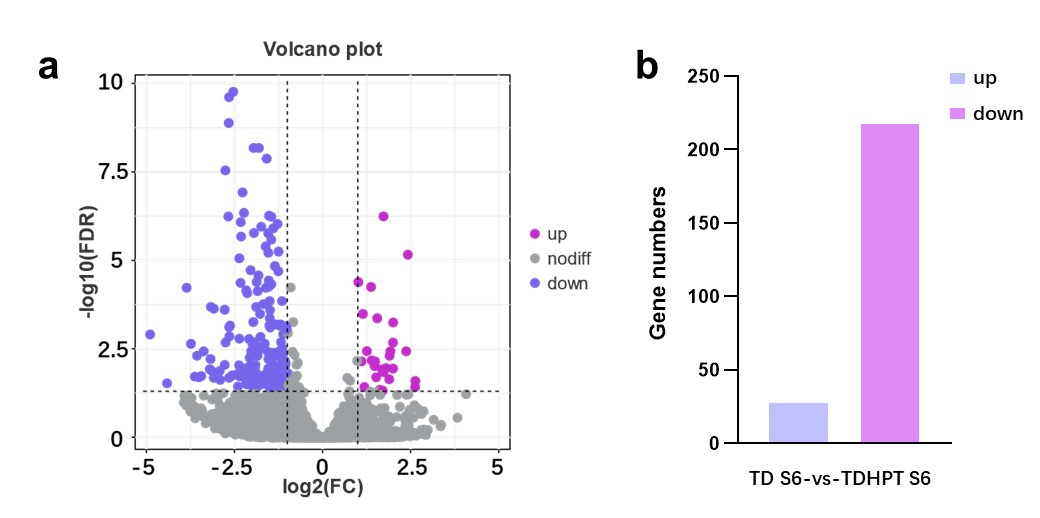


**Figure S12.** Differentially expressed gene (DEG) analysis between TD S6 and TDHPT S6. a) Volcano plot showing the fold change and distribution of DEGs (*n* = 3). b) Bar chart summarizing the number of upregulated and downregulated DEGs in TD S6 compared to TDHPT S6.


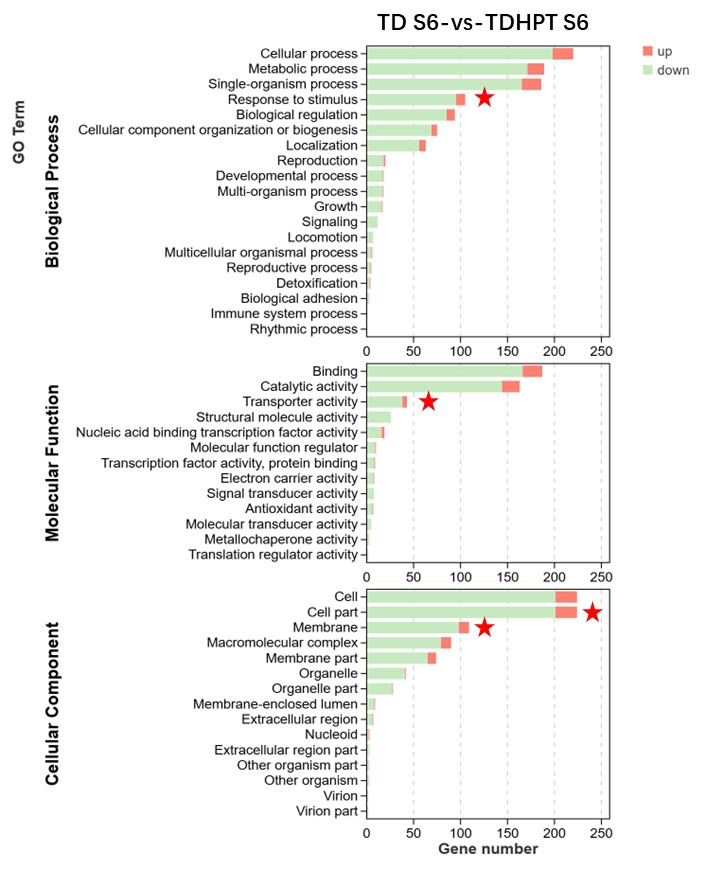


**Figure S13.** GO enrichment analysis of DEGs Between TD S6 and TDHPT S6 (*n* = 3).


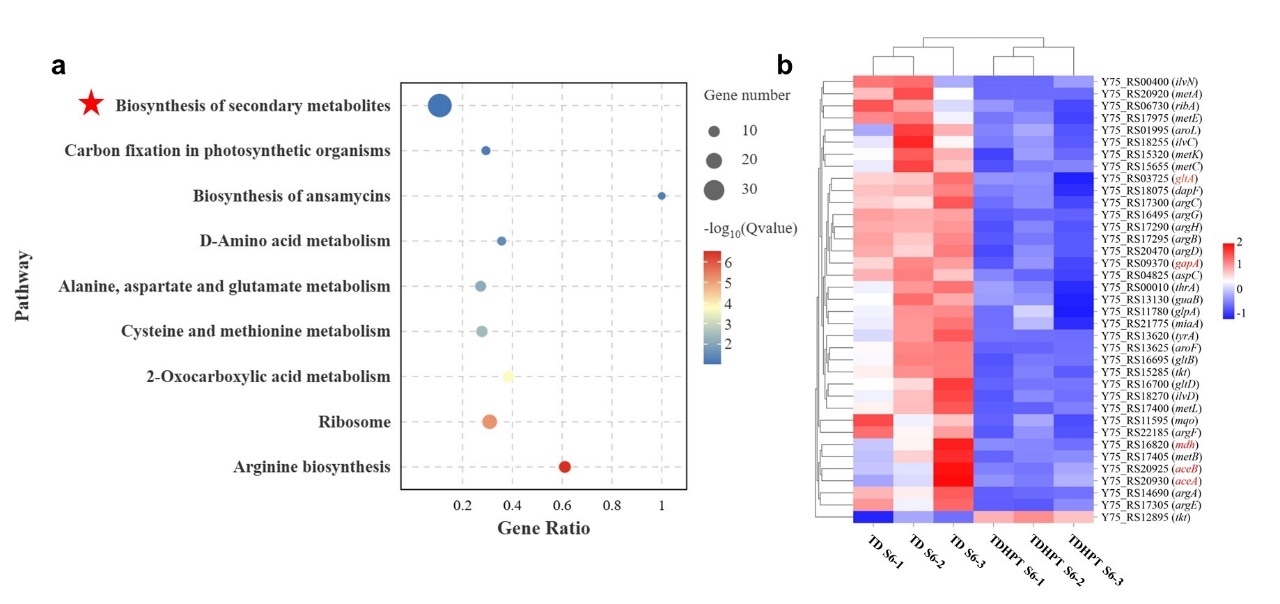


**Figure S14.** KEGG pathway enrichment and gene expression analysis in metabolic Pathways. a) KEGG pathway enrichment bubble chart (*n* = 3). b) Heatmap of gene expression levels in the "Biosynthesis of secondary metabolites" pathway for strains TD S6 and TDHPT S6.

**Table S1.** Strains used in this study.

| Strains | Descriptions | Source |
| --- | --- | --- |
| *E. coli* DH5α | F^-^ supE44, ΔlacU169 (*ϕ80* *lacZ* Δ*M15*), *hsdR17*, *recA1*, *endA1*, *gyrA96*, *thi-1*, *relA1* | Invitrogen |
| *E. coli* W3110 | *E. coli* K-12 derivative, chassis for 3-HP biosynthesis | Lab collection |
| TD | *E. coli* W3110 derivative, Δ*adhE* Δ*pflB* Δ*ldhA* Δ*poxB* Δ*pta-ackA* Δ*yqhD* | This study |
| TD S1 | TD harboring pCOLA-tac-*dhaBCE-gdrAB-glpF* and p15A-trc-*KpydcW* | This study |
| TD S2 | TD harboring pCOLA-tac-*dhaBCE-gdrAB-glpF* and pCOLDF13-trc-*KpydcW* | This study |
| TD S3 | TD harboring pCOLA-tac-*dhaBCE-gdrAB-glpF* and pBR322-trc-*KpydcW* | This study |
| TD S4 | TD harboring pCOLA-tac-*dhaBCE-gdrAB-glpF* and pRSF-trc-*KpydcW* | This study |
| TD S5 | TD harboring p15A-tac-*dhaBCE-gdrAB-glpF* and pCOLDF13-trc-*KpydcW* | This study |
| TD S6 | TD harboring p15A-tac-*dhaBCE-gdrAB-glpF* and pBR322-trc-*KpydcW* | This study |
| TD S7 | TD harboring p15A-tac-*dhaBCE-gdrAB-glpF* and pRSF-trc-*KpydcW* | This study |
| TD S8 | TD harboring pCOLDF13-tac-*dhaBCE-gdrAB-glpF* and pBR322-trc-*KpydcW* | This study |
| TD S9 | TD harboring pCOLDF13-tac-*dhaBCE-gdrAB-glpF* and p15A-RSF-*KpydcW* | This study |
| TD S10 | TD harboring pBR322-tac-*dhaBCE-gdrAB-glpF* and pRSF-trc-*KpydcW* | This study |
| TD MP6ts | TD harboring pMP6ts | This study |
| TD-MUT | TD MP6ts derivative, induced by L-arabinose, cured of plasmid pMP6ts | This study |
| droplet 1 – droplet 50 | TD-MUT derived cultivation in MMC system | This study |
| droplet 2 | The droplet with the highest 3-HP tolerance after 12 days of ALE | This study |
| RAND1 – RAND9 | Droplet 2 derivative, single colonies randomly isolated from droplet 2 | This study |
| HP-LowProd | RAND2 harboring p15A-tac-*dhaBCE-gdrAB-glpF-SENSOR* and pBR322-trc-*KpydcW* | This study |
| HP-MedProd | RAND4 harboring p15A-tac-*dhaBCE-gdrAB-glpF-SENSOR* and pBR322-trc-*KpydcW* | This study |
| HP-HighProd | RAND7 harboring p15A-tac-*dhaBCE-gdrAB-glpF-SENSOR* and pBR322-trc-*KpydcW* | This study |
| HP1 – HP1045 | Droplet 2 derivative, harboring p15A-tac-*dhaBCE-gdrAB-glpF-SENSOR* and pBR322-trc-*KpydcW* | This study |
| HP0032 | The superior strain identified by biosensor-assisted selection | This study |
| TDHPT | HP0032 derivative, cured of plasmids p15A-tac-*dhaBCE-gdrAB-glpF-SENSOR* and pBR322-trc-*KpydcW* | This study |
| TDHPT S6 | TDHPT harboring p15A-tac-*dhaBCE-gdrAB-glpF* and pBR322-trc-*KpydcW* | This study This study |
| TD control | TD harboring pTrc99a | This study |
| glsA^+^ | TD harboring pTrc99a-*glsA* | This study |
| yqjG^+^ | TD harboring pTrc99a-*yqjG* | This study |
| hyaC^+^ | TD harboring pTrc99a-*hyaC* | This study |
| ygdI^+^ | TD harboring pTrc99a-*ygdI* | This study |
| dps^+^ | TD harboring pTrc99a-*dps* | This study |
| cysT^+^ | TD harboring pTrc99a-*cysT* | This study |
| hyaE^+^ | TD harboring pTrc99a-*hyaE* | This study |
| ybaT^+^ | TD harboring pTrc99a-*ybaT* | This study |
| yqjE^+^ | TD harboring pTrc99a-*yqjE* | This study This study |
| slp^+^ | TD harboring pTrc99a-*slp* | This study |
| ynaI^+^ | TD harboring pTrc99a-*ynaI* | This study |
| ompT^-^ | TD derivative, Δ*ompT*, harboring pTrc99a | This study |
| ompF^-^ | TD derivative, Δ*ompF*, harboring pTrc99a | This study |
| btsT^-^ | TD derivative, Δ*btsT*, harboring pTrc99a | This study |
| msbA^-^ | TD derivative, Δ*msbA*, harboring pTrc99a | This study |
| metN^-^ | TD derivative, Δ*metN*, harboring pTrc99a | This study |
| metI^-^ | TD derivative, Δ*metI*, harboring pTrc99a | This study |
| ybjJ^-^ | TD derivative, Δ*ybjJ*, harboring pTrc99a | This study |

**Table S2.** Plasmids used in this study.

| Plasmids | Descriptions | Source |
| --- | --- | --- |
| pCDF-*dhaBCE*-*gdrAB*-*glpF* | ColDF13 ori, Sm^R^, P_T7_-*dhaBCE*-*gdrAB*-*glpF* | Zhang et al.^1^ |
| pRSF-*KpydcW* | RSF ori, Kan^R^, P_T7_-*KpydcW* | Zhang et al.^1^ |
| MP6 | ColDF13 ori, Cm^R^, P_araBAD_, *dhaQ926*, *dam*, *seqA*, *emrR*, *ugi* and *Pmcda1* | Badran and Liu^2^ |
| pCas | pSC101(Ts) ori, Kan^R^, P_araBAD_-RecET, *cas9* | Jiang et al.^3^ |
| pTargetF | pBR322 ori, Sm^R^, P_J23119_-sgRNA | Jiang et al.^3^ |
| pUC57Kan-SENSOR | pUC57 derivative, harboring P_J23109_-*hpTDR* (codon-optimized) and P_hpTD_ | Azenta |
| p15A-tac | p15A ori, Cm^R^, P_tac_ | Lab collection |
| pBR322-trc | pBR322 ori, Kan^R^, P_trc_ | Lab collection |
| pTrc99a | pBR322 ori, Amp^R^, P_trc_, P_lac_-*lacI* | Lab collection |
| pTrc99a | pBR322 ori, Amp^R^, P_trc_-*sfGFP*, P_lac_-*lacI* | Lab collection |
| pORI 1-tac-*dhaBCE*-*gdrAB*-*glpF* | pCDF-*dhaBCE*-*gdrAB*-*glpF* derivative, ORI 1 (ColA, p15A,ColDF13, or pBR322), Cm^R^, P_tac_-*dhaBCE*-*gdrAB*-*glpF* | This study |
| pORI 2-trc-*KpydcW* | pRSF-*KpydcW* derivative, ORI 2 (p15A, ColDF13, pBR322, RSF), Kan^R^, P_tac_-*KpydcW* | This study |
| pMP6ts | MP6 derivative, pSC101(tS) ori, Kan^R^ | This study |
| pSEN T0 | p15A ori, Cm^R^, P_J23109_-*hpTDR*, P*_hpTD_*-*sfGFP* | This study |
| pSEN POSI | pSEN T0 derivative, without *hTDR* | This study |
| pSEN NEGA | pSEN T0 derivative, without *sfGFP* | This study |
| pSEN T1 | pSEN T0 derivative, P_J23109_-B0031-*hpTDR*, P*_hpTD_*-B0031-*sfGFP* | This study |
| pSEN T2 | pSEN T0 derivative, P_J23109_-B0031-*hpTDR*, P*_hpTD_*-B0032-*sfGFP* | This study |
| pSEN T3 | pSEN T0 derivative, P_J23109_-B0031-*hpTDR*, P*_hpTD_*-B0030-*sfGFP* | This study |
| pSEN T4 | pSEN T0 derivative, P_J23109_-B0032-*hpTDR*, P*_hpTD_*-B0031-*sfGFP* | This study |
| pSEN T5 | pSEN T0 derivative, P_J23109_-B0032-*hpTDR*, P*_hpTD_*-B0032-*sfGFP* | This study |
| pSEN T6 | pSEN T0 derivative, P_J23109_-B0032-*hpTDR*, P*_hpTD_*-B0030-*sfGFP* | This study |
| pSEN T7 | pSEN T0 derivative, P_J23109_-B0030-*hpTDR*, P*_hpTD_*-B0031-*sfGFP* | This study |
| pSEN T8 | pSEN T0 derivative, P_J23109_-B0030-*hpTDR*, P*_hpTD_*-B0032-*sfGFP* | This study |
| pSEN T9 | pSEN T0 derivative, P_J23109_-B0030-*hpTDR*, P*_hpTD_*-B0030-*sfGFP* | This study |
| p15A-tac-*dhaBCE*-*gdrAB*-*glpF-SENSOR* | p15A-tac-*dhaBCE*-*gdrAB*-*glpF* derivative, harboring biosensor element | This study |
| pELIM-pBR322 | pSC101(Ts) ori, Amp^R^, P_lacI_-sgRNA^pBR322^, *cas9* | This study |
| pELIM-15A | pELIM-pBR322 derivative, P_lacI_-sgRNA^p15A^ | This study |
| pTrc99a-*glsA* | pTrc99a derivative, harboring P*_lacIq_*-*glsA* | This study |
| pTrc99a-*glsA* | pTrc99a derivative, harboring P*_lacIq_*-*yqjG* | This study |
| pTrc99a-*glsA* | pTrc99a derivative, harboring P*_lacIq_*-*hyaC* | This study |
| pTrc99a-*glsA* | pTrc99a derivative, harboring P*_lacIq_*-*ygdI* | This study |
| pTrc99a-*glsA* | pTrc99a derivative, harboring P*_lacIq_*-*dps* | This study |
| pTrc99a-*glsA* | pTrc99a derivative, harboring P*_lacIq_*-*cysT* | This study |
| pTrc99a-*glsA* | pTrc99a derivative, harboring P*_lacIq_*-*hyaE* | This study |
| pTrc99a-*glsA* | pTrc99a derivative, harboring P*_lacIq_*-*ybaT* | This study |
| pTrc99a-*glsA* | pTrc99a derivative, harboring P*_lacIq_*-*yqiE* | This study |
| pTrc99a-*glsA* | pTrc99a derivative, harboring P*_lacIq_*-*slp* | This study |
| pTrc99a-*glsA* | pTrc99a derivative, harboring P*_lacIq_*-*ynaI* | This study |
| pTargetF-*ompT* | pTargetF derivative, harboring P_J23119_-sgRNA*^ompT^* | This study |
| pTargetF-*ompF* | pTargetF derivative, harboring P_J23119_-sgRNA*^ompF^* | This study |
| pTargetF-*btsT* | pTargetF derivative, harboring P_J23119_-sgRNA*^btsT^* | This study |
| pTargetF-*bsbA* | pTargetF derivative, harboring P_J23119_-sgRNA*^bsbA^* | This study |
| pTargetF-*metN* | pTargetF derivative, harboring P_J23119_-sgRNA*^metN^* | This study |
| pTargetF-*metI* | pTargetF derivative, harboring P_J23119_-sgRNA*^metI^* | This study |
| pTargetF-*ybjJ* | pTargetF derivative, harboring P_J23119_-sgRNA*^ybjJ^* | This study |

**Table S3.** Primers used in this study.

| Primers | Sequences（5ʹ-3ʹ） |
| --- | --- |
| backbone1 F | cattatacgagccgatgattaattgt |
| backbone1 R | ctgccaccgctgagcaataa |
| DGF F | TTAATCATCGGCTCGTATAATGtttaactttaataaggagatataccatg |
| DGF R | TTATTGCTCAGCGGTGGCttacagcgaagctttttgttct |
| backbone2 F | gaagatcatcttattaatcagataaaatat |
| backbone2 R | aaaaacggctttgccgc |
| Ori1-ColA F | TATCTGATTAATAAGATGATCTTCtggtgtcgggaatccgta |
| Ori1-ColA R | GCGGCAAAGCCGTTTTTaaacgtcctagaagatgc |
| Ori1-ColDF13 F | TATCTGATTAATAAGATGATCTTCgatcaaaggatcttcttgagat |
| Ori1-ColDF13 R | GCGGCAAAGCCGTTTTTgcgctgcggacacatac |
| Ori1-pBR322 F | TATCTGATTAATAAGATGATCTTCttgagatcctttttttctgcgcg |
| Ori1-pBR322 R | GCGGCAAAGCCGTTTTTtttccataggctccgccc |
|  | For the construction of plasmids designed to express the *tac-dhaBCE-gdrAB-glpF* cassette |
| backbone3 F | cattatacgagccggatga |
| backbone3 R | ggctgttttggcggatgag |
| *KpydcW* F | aaatcgagcacataaggagat |
| *KpydcW* R | ATCCGCCAAAACAGCCtcagctatgcttaaccatcac |
| backbone4 F | AATCATCCGGCTCGTATAATGgaagatcctttgatcttttctac |
| backbone4 R | aacgccagcaacgcg |
| Ori2-p15A F | GAAAAGATCAAAGGATCTTCttgagatcgttttggtctgc |
| Ori2-p15A R | GCGTTGCTGGCGTTtttccataggctccgccc |
| Ori2-ColDF13 F | GAAAAGATCAAAGGATCTTCgatcaaaggatcttcttgagat |
| Ori2-ColDF13 R | GCGTTGCTGGCGTTgcgctgcggacacatac |
| Ori2-RSF F | GAAAAGATCAAAGGATCTTCaacggaatagctgttcgtt |
| Ori2-RSF R | GCGTTGCTGGCGTTcttccgcttcctcgctc |
|  | For the construction of plasmids designed to express the trc-*KpydcW* cassette |
| KanR-TS F | ACATTTGAAGAGATAAATTGCACTGAAATtgcagggaaagccacgtt |
| KanR-TS R | GCTTTTGACTACATGCCCATGatggacagttttccctttgat |
|  | For the construction of plasmid pMP6ts |
| SENSOR ELE F | GTAGCACCTGAAGTCAGCttagcccaggcgatacgg |
| SENSOR ELE R | CTCCTTCTTCTGTTTGGCtgggctacctttataagcagattaaa |
| *sfGFP* F | CCAAACAGAAGAAGGAGATAATTTatgcgtataggtgaagaactgttca |
| *sfGFP* R | TGGTTTACCGGTTTATTGACagaaacgcaaaaaggccat |
| p15A F | gctgacttcaggtgctac |
| CmR R | gtcaataaaccggtaaaccagca |
|  | For the construction of plasmid pSEN T0 |
| backbone5 F | AAAAAAGGAGCATCATCAgctgacttcaggtgctac |
| backbone5 R | tgatgatgctccttttttacaggtt |
|  | For the construction of plasmid pSEN POSI |
| backbone6 F | AGAAGAAGGAGATAATTTcgagagtagccaactgcc |
| backbone6 R | aaattatctccttcttctgttt |
|  | For the construction of plasmid pSEN NEGA |
| RBS1-*hpTDR* F | TACTTTCCTGTGTGACTCTAGAgctagcacagtccctaggactga |
| RBS1-*hpTDR* R | GAGTCACACAGGAAAGTACTAGatgcaagaaaacagtgcgctg |
| RBS2-*hpTDR* F | GGTTTCCTGTGTGAACTCTAGAgctagcacagtccctaggactga |
| RBS2-*hpTDR* R | GAGTTCACACAGGAAACCTACTAGatgcaagaaaacagtgcgctg |
| RBS3-*hpTDR* F | TTCTCCTCTTTAATCTCTAGAgctagcacagtccctaggactga |
| RBS3-*hpTDR* R | GAGATTAAAGAGGAGAATACTAGatgcaagaaaacagtgcgctg |
| RBS1-*sfGFP* F | GAGTCACACAGGAAAGTACTAGatgcgtataggtgaagaactgttca |
| RBS1-*sfGFP* R | TACTTTCCTGTGTGACTCTAGAtgggctacctttataagcagattaaa |
| RBS2-*sfGFP* F | GAGTTCACACAGGAAACCTACTAGatgcgtataggtgaagaactgttca |
| RBS2-*sfGFP* R | GGTTTCCTGTGTGAACTCTAGAtgggctacctttataagcagattaaa |
| RBS3-*sfGFP* F | GAGATTAAAGAGGAGAATACTAGatgcgtataggtgaagaactgttca |
| RBS3-*sfGFP* R | TTCTCCTCTTTAATCTCTAGAtgggctacctttataagcagattaaa |
|  | For the construction of biosensor plasmid incorporating various combinations of RBSs |
| SENSOR F | GTGGCAGCAGCCTAGGTTAcggtttattgacagaaacgc |
| SENSOR R | AGCCTCCGGTCGGAGGCTTTTGACTttagcccaggcgatacggc |
| backbone7 F | taacctaggctgctgccacc |
| backbone7 R | AGCCTCCGACCGGAGGCTTTTGACTatttcgattatgcggccgt |
|  | For the construction of plasmid p15A-tac-*dhaBCE-gdrAB-glpF-*SENSOR |
| AmpR F | ATAAACAGTAATACAAGGGGTGTTatgagtattcaacatttccgtgt |
| AmpR R | TTCCAACCAATTAACCAATTCTGAttaccaatgcttaatcagtgaggc |
| pSC101(ts) F | tcagaattggttaattggttggaa |
| sgRNA R | ACATTTCCCCGAAAAGTGCCAcgcatcctcacgataatatcc |
| *cas9* F | aacaccccttgtattactgtttat |
| *cas9* R | tggcacttttcggggaaatg |
|  | For the construction of plasmid pELIM-pBR322 |
| sgRNA-p15A F | ctcctccaagccagttacctGTTTTAGAGCTAGAAATAGCAA |
| sgRNA-p15A R | aggtaactggcttggaggagTGTGTGAAATTGTTATCCGC |
|  | For the construction of plasmid pELIM-p15A |
| pTrc99a F | attcaccaccctgaattgact |
| pTrc99a R | gcgcaacgcaattaatgt |
| *glsA* F | CAATTCAGGGTGGTGAATatgttagatgcaaacaaattacagcag |
| *glsA* R | ACATTAATTGCGTTGCGCtcagcccttaaacacgttatagcc |
| *yqjG* F | CAATTCAGGGTGGTGAATatgggtcaactgattgacggc |
| *yqjG* R | ACATTAATTGCGTTGCGCttaaccgaagcgaacatctcg |
| *hyaC* F | CAATTCAGGGTGGTGAATatgcaacagaaaagcgacaac |
| *hyaC* R | ACATTAATTGCGTTGCGCtcatgaacgctccttgttact |
| *ygdI* F | CAATTCAGGGTGGTGAATatgaaaaagactgccgcaatt |
| *ygdI* R | ACATTAATTGCGTTGCGCttactgatccagttcgaccat |
| *dps* F | CAATTCAGGGTGGTGAATatgagtaccgctaaattagttaaatca |
| *dps* R | ACATTAATTGCGTTGCGCttattcgatgttagactcgataaacca |
| *cysT* F | CAATTCAGGGTGGTGAATatgtttgctgtctcctccaga |
| *cysT* R | ACATTAATTGCGTTGCGCttaatgacctaccacacgccg |
| *hyaE* F | CAATTCAGGGTGGTGAATatgagcaacgacacgccattt |
| *hyaE* R | ACATTAATTGCGTTGCGCtcatgaggcacgctcctgctg |
| *ybaT* F | CAATTCAGGGTGGTGAATatgatgaacacggaaggtaat |
| *ybaT* R | ACATTAATTGCGTTGCGCttatacggttttattgcgctt |
| *yqjE* F | CAATTCAGGGTGGTGAATatggcggacactcatcacgca |
| *yqjE* R | ACATTAATTGCGTTGCGCttactgctcacgggactcctc |
| *slp* F | CAATTCAGGGTGGTGAATatgaacatgacaaaaggtgca |
| *slp* R | ACATTAATTGCGTTGCGCttatttgaccagctcaggtgt |
| *ynaI* F | CAATTCAGGGTGGTGAATatgatcgctgaactgtttaca |
| *ynaI* R | ACATTAATTGCGTTGCGCttagcgaccctgttccggcgg |
|  | For the construction of plasmid designed to overexpress *glsA*, *yqjG*, *hyaC*, *ygdI*, *dps*, *cysT*, *hyaE*, *ybaT*, *yqjE*, *slp*, or *ynaI* |
| sgRNA-*ompT* F | GAAAAGTCAGTCAACTCGACgttttagagctagaaatagc |
| sgRNA*-ompT* R | GTCGAGTTGACTGACTTTTCactagtattatacctaggac |
| *ompT*-up F | gtcacagagtgtcgtatgcg |
| *ompT*-up R | TTGGCGTTCTTAcataaaagttctccattcaatcgtt |
| *ompT*-down F | GGAGAACTTTTATGtaagaacgccaactaaaatttccc |
| *ompT*-down R | gactcacactccctttggt |
|  | For the knockout of *ompT* |
| sgRNA-*ompF* F | GAAAACAGTTACGGTGGCAAgttttagagctagaaatagc |
| sgRNA*-ompF* R | TTGCCACCGTAACTGTTTTCactagtattatacctaggac |
| *ompF*-up F | tggttttgaacgtctgattgc |
| *ompF*-up R | AGGTGTGCTATTAcattatttattaccctcatggtt |
| *ompF*-down F | TAATAAATAATGtaatagcacacctctttgtt |
| *ompF*-down R | gcctgagcttcattcaggct |
|  | For the knockout of *ompF* |
| sgRNA-*btsT* F | GATCGTGGTCGCCTCTGTATgttttagagctagaaatagc |
| sgRNA*-btsT* R | ATACAGAGGCGACCACGATCactagtattatacctaggac |
| *btsT*-up F | ccgcccagtttttttccgc |
| *btsT*-up R | CTAAACACGGTTAcatagtaaaacctggcatgta |
| *btsT*-down F | GGTTTTACTATGtaaccgtgtttagccccgc |
| *btsT*-down R | cgatggtgaactggttcatctgc |
|  | For the knockout of *btsT* |
| sgRNA-*msbA* F | TATTACCTACGATTCCGAACgttttagagctagaaatagc |
| sgRNA*-msbA* R | GTTCGGAATCGTAGGTAATAactagtattatacctaggac |
| *msbA*-up F | caacaactgattatcccctggc |
| *msbA*-up R | ATTTTTTCGATCAcattcaaaaaaccagcattt |
| *msbA*-down F | TGGTTTTTTGAATGtgatcgaaaaaatctggtctggt |
| *msbA*-down R | ccactacatgttcaagctgagca |
|  | For the knockout of *msbA* |
| sgRNA-*metN* F | GTGCCAGCTGGACAAATTTAgttttagagctagaaatagc |
| sgRNA*-metN* R | TAAATTTGTCCAGCTGGCACactagtattatacctaggac |
| *metN*-up F | tccgccgcgttttctgct |
| *metN*-up R | ATCATCGGCTCAcattgattatttattatcgtcattaagt |
| *metN*-down F | AAATAATCAATGtgagccgatgatgtggct |
| *metN*-down R | ttgaacgccataccttattcct |
|  | For the knockout of *metN* |
| sgRNA-*metI* F | CCGTTTACCCGCGTTATTGTgttttagagctagaaatagc |
| sgRNA*-metI* R | ACAATAACGCGGGTAAACGGactagtattatacctaggac |
| *metI*-up F | caccgaaagacgaggtcaa |
| *metI*-up R | TGTTGAACGTTActcagacataacccagtacc |
| *metI*-down F | GTTATGTCTGAGtaacgttcaacacaacataaataatt |
| *metI*-down R | aaagataccgtctttcgccg |
|  | For the knockout of *metI* |
| sgRNA-*ybjJ* F | CTCTGTCTCGATCGCTGAAAgttttagagctagaaatagc |
| sgRNA*-ybjJ* R | TTTCAGCGATCGAGACAGAGactagtattatacctaggac |
| *ybjJ*-up F | cccattttgtggagcccat |
| *ybjJ*-up R | TAATGCTCATCAcatagatcacgtcaaaataagaaga |
| *ybjJ*-down F | GACGTGATCTATGtgatgagcattaaattaattgcggta |
| *ybjJ*-down R | ccattgcaaaactaaagcct |
|  | For the knockout of *ybjJ* |
| Q-*recA* F | attctacgcctctgttcgtct |
| Q-*recA* R | cttttacgcccaggtcaacca |
| Q-*gapA* F | agttgacctgaccgttcgtct |
| Q-*gapA* R | agcatcgaacacggaagtgcaa |
| Q-*gltA* F | gggcaatcttcataccgtcac |
| Q-*gltA* R | aagaaactgtacccgaacgtcg |
| Q-*mdh* F | cttcaacgtaggcacattcgac |
| Q-*mdh* R | gctgatctgaccaaacgcatc |
| Q-*aceA* F | tccgcgctatgtcgattacttcc |
| Q-*aceA* R | ctttgccgcccatgtgacc |
| Q-*aceB* F | attcactggcaccagact |
| Q-*aceB* R | ttttccggcaagtgcagac |
|  | Primers for qPCR verification |
